# Substrate recognition principles for the PP2A-B55 protein phosphatase

**DOI:** 10.1126/sciadv.adp5491

**Published:** 2024-10-02

**Authors:** Thomas Kruse, Dimitriya H. Garvanska, Julia K. Varga, William Garland, Brennan C. McEwan, Jamin B. Hein, Melanie Bianca Weisser, Iker Benavides-Puy, Camilla Bachman Chan, Paula Sotelo-Parrilla, Blanca Lopez Mendez, A. Arockia Jeyaprakash, Ora Schueler-Furman, Torben Heick Jensen, Arminja N. Kettenbach, Jakob Nilsson

**Affiliations:** ^1^ Novo Nordisk Foundation Center for Protein Research, Faculty of Health and Medical Sciences, Blegdamsvej 3B, 2200 Copenhagen, Denmark.; ^2^ Department of Microbiology and Molecular Genetics, Institute for Biomedical Research Israel-Canada, Faculty of Medicine, The Hebrew University of Jerusalem, Jerusalem 9112001, Israel.; ^3^ Department of Molecular Biology and Genetics, Aarhus University, Universitetsbyen 81, Aarhus, Denmark.; ^4^ Biochemistry and Cell Biology, Geisel School of Medicine at Dartmouth College, Hanover, NH, USA.; ^5^ Gene Center Munich, Ludwig-Maximilians–Universität München, Munich 81377, Germany.; ^6^ Wellcome Centre for Cell Biology, University of Edinburg, Edinburgh EH9 3BF, UK.

## Abstract

The PP2A-B55 phosphatase regulates a plethora of signaling pathways throughout eukaryotes. How PP2A-B55 selects its substrates presents a severe knowledge gap. By integrating AlphaFold modeling with comprehensive high-resolution mutational scanning, we show that α helices in substrates bind B55 through an evolutionary conserved mechanism. Despite a large diversity in sequence and composition, these α helices share key amino acid determinants that engage discrete hydrophobic and electrostatic patches. Using deep learning protein design, we generate a specific and potent competitive peptide inhibitor of PP2A-B55 substrate interactions. With this inhibitor, we uncover that PP2A-B55 regulates the nuclear exosome targeting (NEXT) complex by binding to an α-helical recruitment module in the RNA binding protein 7 (RBM7), a component of the NEXT complex. Collectively, our findings provide a framework for the understanding and interrogation of PP2A-B55 function in health and disease.

## Teaser

α helices in PP2A-B55 substrates bind a conserved pocket on B55 through a common mechanism that is conserved in eukaryotes.

## INTRODUCTION

Dynamic phosphorylation of Ser/Thr residues is central for modulating protein activities throughout eukaryotes (*1*, *2*). This regulatory mechanism depends on the selective recognition of substrates and substrate residues by kinase and phosphatase enzymes. While the basis for kinase specificity has been extensively characterized (*3*, *4*), the parameters mediating protein phosphatase specificity are less well understood. In recent years, it has become clear that members of the Ser/Thr phosphoprotein phosphatases (PPPs; PP1 to PP7) achieve specificity by binding to short linear motifs (SLiMs) in the intrinsically disordered regions of their substrates or substrate specifiers (*5*–*7*). SLiMs bind to defined pockets distal from the active site on the phosphatase catalytic subunits. However, whether SLiMs mediate substrate recognition by all PPP members is not known.

The PPP PP2A regulates numerous fundamental signaling pathways and is deregulated in human diseases underscoring the importance of establishing fundamental principles of PP2A substrate recognition (*8*–*10*). PP2A holoenzymes are trimeric and composed of a catalytic subunit (PPP2CA/B), a scaffolding subunit (PPP2R1A/B), and one of several regulatory B subunits present in multiple isoforms (B55α-δ, B56α-ε, PR72/PR130, and STRN1 to STRN4) (*6*, *7*, *11*). The B55 and B56 regulatory subunits are fully conserved from yeast to human, acting as major determinants of PP2A holoenzyme specificity. We and others previously uncovered a conserved binding motif for PP2A-B56, the so called LxxIxE motif, that engages a highly conserved binding pocket on the B56 regulatory subunit (*12*–*14*). This finding provided important insight into PP2A-B56 regulation of signaling and opened up possibilities for the precise engineering of signaling pathways.

In contrast to PP2A-B56, we have a limited understanding of PP2A-B55 substrate recognition. PP2A-B55 is a key regulator of multiple cellular processes such as the cell cycle, where it antagonizes cyclin-dependent kinase activity. In turn, PP2A-B55 activity is tightly regulated by the inhibitory ENSA/ARPP19 and FAM122A proteins, ensuring proper cell cycle execution (*15*–*20*). ENSA/ARPP19 is phosphorylated by the MASTL kinase in human cells, which is required for their inhibitory activity toward PP2A-B55, while FAM122A seems to be a constitutive inhibitor. Recently, the p107 protein, a retinoblastoma-related tumor suppressor also associated with cell cycle regulation and a substrate of PP2A-B55, was proposed to bind B55 via SLiM (*21*), while modeling and cryo–electron microscopy (cryo-EM) structures of the B55 inhibitors ARPP19 and FAM122A revealed binding of α helices to B55 (*20*, *22*, *23*). It is unclear whether substrates bind to B55 via SLiMs, while inhibitors use a distinct mechanism.

Here, we have combined AlphaFold2 (AF2) modeling (*24*) with experimental validation, which identified B55-interacting α helices in numerous proteins conferring substrate specificity to PP2A-B55. On the basis of our structural models, we have furthermore used ProteinMPNN (*25*) to design a strong and highly specific B55 inhibitor and used it to demonstrate PP2A-B55 roles in cell division and RNA degradation. Our findings have important implications for understanding and engineering PP2A signaling networks and provide an example of an extended structured binding element conferring specificity to a PP2A-like family member.

## RESULTS

### A conserved pocket on the B55 subunit binds α helices in interactors

**Fig. 1.**
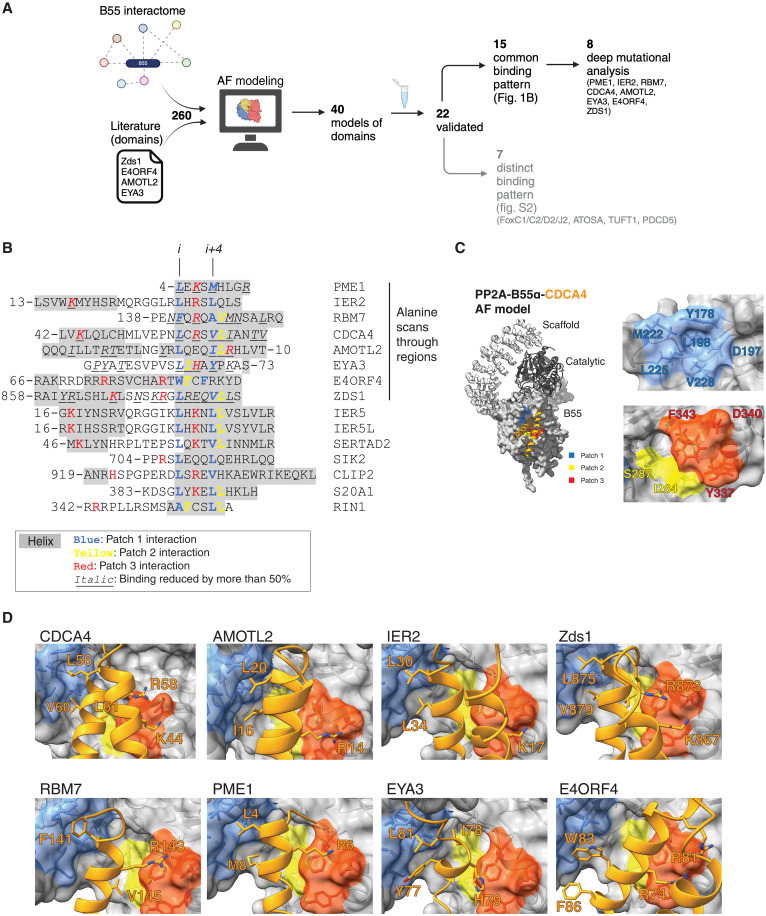
Helical motifs engage a conserved binding pocket on PP2A-B55. (**A**) Schematic of pipeline to identify PP2A-B55 binding elements. Generated with BioRender. (**B**) Alignment of validated instances with helices in gray and residues contacting patch 1 in blue, patch 2 in yellow, and patch 3 in red. The first eight proteins on the list were the proteins that were scanned by single alanine mutagenesis, and residues found to reduce binding by at least 50% upon mutation to alanine are in italic. (**C**) Model of the PP2A-B55-CDCA4 complex with the different patches in B55 indicated in different colors. (**D**) AF2 models of the indicated proteins and their interaction with B55. Core residues forming interactions in the different patches are presented as sticks. AF, AlphaFold.

Despite the considerable amount of structural and biochemical information available on PP2A-B55, a key unresolved question is the molecular basis for its substrate recognition. To address this question, we established a discovery pipeline that integrates AF2 multimer modeling with experimental high-resolution mutagenesis scanning of PP2A-B55 interacting proteins (Fig. 1A). AF2 can not only generate highly accurate models but also provides confident measures of the model quality (*24*, *26*–*28*). As input for the pipeline, we used our previous mass spectrometry (MS) interactome data that identified 256 protein interactors specific for PP2A-B55α (*14*, *29*). We expanded this with reported B55 binding domains from the literature (EYA3 and AMOTL2) as well as instances from yeast (Zds1) and viruses (E4ORF4) (*30*–*34*). Applying AF2 using B55α on this set of interactors returned 40 models with good confidence scores [median predicted aligned error at the interface (iPAE) < 10 and average interface local distance difference test (pLDDT) measuring local confidence > 70 (fig. S1A, table S1, and dataset S1)]. The values were calculated over the conserved B55α binding site (see Materials and Methods and fig. S1B). Although below the cutoff, we included EYA3 and AMOTL2 because of previous validation of modeled binding domains in the literature (*30*, *31*). Most of the models revealed defined binding elements predicted to engage a fully conserved surface on B55 present in all isoforms that has previously been implicated in substrate binding (fig. S1B) (*19*, *21*, *35*). The recently reported binding elements in FAM122A and cAMP-regulated phosphoprotein 19 (ARPP19)/alpha-endosulfine (ENSA), which were not part of the training set for AF2, were also identified through this approach (*20*, *22*, *23*). The AF2 models for FAM122A and ARPP19 overlap at the atomic level with the experimental structure for FAM122A, as well as for the helix covering the L49/L53 motif in ARPP19, providing confidence in our models (fig. S1, C and D). During our work, the cryo-EM structure of IER5 bound to PP2A-B55 was reported, which overall resembles our AF2 model, further supporting our conclusions (*36*).

Of the predicted 40 binding elements, we experimentally validated 19 instances using immunopurifications from HeLa cells (figs. S2 to S10). To this list, we added three additional instances based on high sequence homology [SERTAD2 (SERTA domain), IER5, and IER5L similar to IER2], collectively expanding the validated and modeled B55α binding elements to 22.

We inspected the 22 AF2 models for common features, which revealed that all of them used α-helical elements to bind B55α. The presence of instances in yeast, humans, and viruses suggests an evolutionary conserved binding mechanism. Consistently, SERTA domains from numerous species spanning close to a billion years of evolution were observed to bind PP2A-B55α (fig. S11). For 15 binding elements, we identified a common pattern of binding with either a single helix (PME1, RBM7, EYA3, SIK2, S20A1, and RIN1) or helix-loop-helix (CDCA4, SERTAD2, IER2/5/5 L, Zds1, CLIP2, and AMOTL2) engaging B55α (Fig. 1, B to D). E4ORF4 also used this binding mechanism through a three-helix bundle. In all cases, a single helix, the binding helix, binds to B55α in a similar manner. In the case of helix-loop-helix binders and E4ORF4, additional contacts with B55α are established via the preceding additional helix (or helices, in the case of E4ORF4) (fig. S10). We also compared AF2 predictions to that of AF3 and RosettaFold for CDCA4, which generated identical models (fig. S11).

Despite limited sequence similarity, our structural analysis uncovered general principles for how the binding helix engages B55α. These principles involve a conserved contact of a central motif at the start of the binding helix with an adjacent hydrophobic pocket composed of B55α residues Y178, D197, M222, L225, and V228 that we term patch 1 (Fig. 1, B to D). This central motif involves primarily hydrophobic residues at positions *i* and *i + 4* in the helix (e.g., L56/V60 in CDCA4). This patch is complemented by patch 2, composed of B55α residues I284 and S287, which is contacted by residues at position *i* + 1 and/or *i* + 5 that can be hydrophobic (such as L61 in CDCA4A). Patch 3 is centered around B55α residue D340 that forms electrostatic interactions predominantly with the residue located on the opposite side of the binding helix, at position *i +* 2 (e.g., R58 in CDCA4). In the helix-loop-helix type of binders, the *i +* 2 position is supplemented by additional positively charged residues (e.g., K44 in the adjacent helix of CDCA4). The interactions with patch 3 display more variation and may involve residues contributed from different parts of the substrate, e.g., in Zds1 where residue *i* + 2 (E27) is negatively charged and R23 in a nearby loop forms the salt bridge with D340 instead. Besides these two patches, additional contacts are formed with the side of the binding pocket involving aromatic B55α residues Y337 and F343. Although our AF2 models support these general principles of binding, they also revealed distinct additional contacts to B55α for each binder. The AF2 models of 7 of our 22 validated instances (FOXC1/C2/D2/J2, ATOSA, TUFT1, and PDCD5) suggested that they bound B55α through α-helical structures contacting patches 1 to 3 (fig. S2). However, these seven instances contacted B55α in a more heterogeneous manner not readily conforming to the common binding patterns observed above, and further work is needed to experimentally validate these models.

To obtain experimental support for the AF2 models, we expressed and immunoprecipitated yellow fluorescent protein (YFP)–tagged interactors from HeLa cells and monitored binding to PP2A-B55α by Western blot. Using this approach, we conducted comprehensive biochemical mappings involving truncation analysis, and 10 and/or 5 alanine walks through several interactors mapping regions of interaction consistent with the AF2 models (figs. S3 to S5 and S7 to S9). To further identify specific amino acid binding determinants at the B55α-interactor interface, single alanine mutational scans of these interfaces were performed for CDCA4, IER2, RBM7, AMOTL2, EYA3, PME1, Zds1, and E4ORF4 (Fig. 1B and figs. S3 to S10). For the majority of instances, the contribution of key amino acid residues interacting with the different patches of B55α as modeled by AF2 was confirmed (Fig. 1B), and they are generally in agreement with computational alanine scanning performed on these models (table S2 and see Materials and Methods). However, for a few instances such as IER2 single amino acid mutation of L30 and L34 contacting patch 1 did not affect binding, while mutating S33 to S37 abolished binding, suggesting that multiple mutations at this contact point are needed to prevent the interaction. In contrast, the mutation of IER2 K17 contacting patch 3 prevented the interaction, indicating a large contribution of this residue to the interaction. For the viral substrate E4ORF4, the interaction is in addition dominated by strong electrostatic effects, which might explain why single alanine mutations are insufficient to disrupt binding to B55α. Through MS analysis, we confirmed that the binding of several isoforms of B55 was abolished when we mutated key binding residues in CDCA4, IER2, PME1, AMOTL2, and RBM7 in line with the conservation of the B55 binding pocket (fig. S12 and table S3). This argues that the identified binding mechanism is shared by all B55 isoforms.

**Fig. 2.**
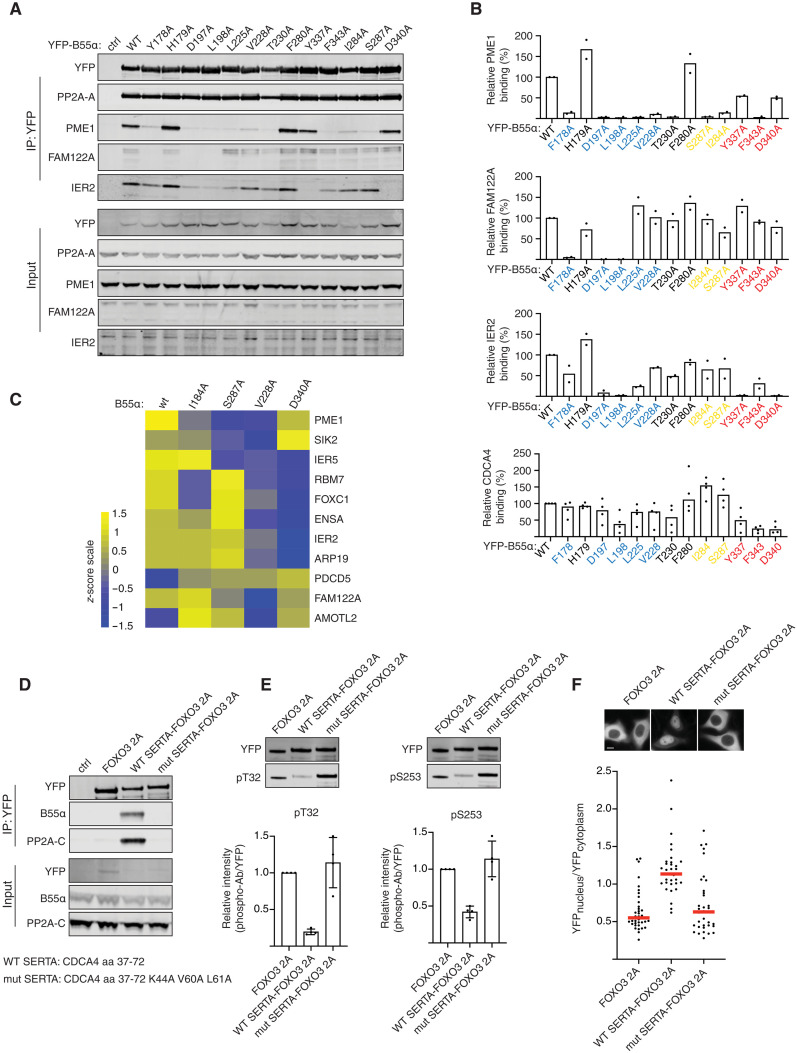
Helical motifs can act as substrate specifying elements. (**A**) Indicated B55 mutants were purified and binding to IER2, PME1, CDCA4, and FAM122A determined and quantified. (**B**) Quantifications of relative binding of indicated proteins to B55 of two or four biological replicates and the averages are shown. (**C**) Heatmap illustrating the changes in binding pattern of the indicated proteins to the different B55 variants as determined by MS. The scale is log 2. *n* = 4 technical replicates. (**D**) CDCA4 WT or mutant SERTA domain was fused to FOXO3 2A, and binding to PP2A-B55 was monitored by Western blot. (**E**) As in (D) but immunoprecipitations (IPs) probed with FOXO3 phospho-specific antibodies as indicated. *n* = 3 biological replicates. Error bars are mean with SD. (**F**) Subcellular localization of FOXO3 fusion proteins by live-cell microscopy. Shown is a representative experiment of three independent experiments. Each dot represents a cell analyzed. Median is indicated with red line. Scale bar, 10 μM.

To investigate the contribution of the different B55α patches, we analyzed the binding of PME1, FAM122A, IER2, and CDCA4 to a panel of B55α mutants covering the different patch residues (Fig. 2, A and B; fig. S13; and table S2). This revealed substantial diversity of these proteins in their binding requirements for the three patches. PME1 required amino acids in all three patches for binding, whereas only residues in patch 1 seemed necessary for FAM122A. IER2 and CDCA4 shared similar binding characteristics with requirements from residues in patch 1 and patch 3. To explore whether this patch variability is a general phenomenon shared with other B55α interactors, we performed MS analysis of B55α WT, V228A, I284A, S287A, and D340A immunopurifications Fig. 2B. The results confirmed on a global scale that B55α interactors have differences in dependencies for the different patches on B55α despite common features of the binding mechanism (Fig. 2C and fig. S13). This is exemplified by IER2 versus IER5 binding to B55α. Despite their almost identical N-terminal helix-loop-helix structure, IER5 requires S287 in patch 2 for efficient interaction, whereas IER2 does not (Fig. 2C).

In summary, our AF2 models and experimental data collectively support that α helices in binders share key amino acid signatures that differentially engage discrete hydrophobic and electrostatic patches on B55α, making it a remarkably versatile platform for mediating protein-protein interactions.

### Helical binding elements confer substrate specificity to PP2A-B55

An important outstanding question was whether the α helices acted as substrate specifiers or inhibitors of PP2A-B55. Our MS analysis revealed that a number of phosphorylation sites in RBM7 (fig. S5A), PME1 (fig. S6A), and AMOTL2 (fig. S8A) had a higher degree of occupancy when we mutated the B55α binding helix, which would support a role in substrate specification (table S3). To directly test this, we engrafted the SERTA domain (amino acid residues 37 to 72) from CDCA4 or a mutant variant (SERTA K44A/V60A/L61A) onto the forkhead box protein O3 (FOXO3) transcription factor on which we mutated its endogenous PP2A-B56 binding site (FOXO3 2A) (*14*). We previously showed that PP2A-B56 regulates FOXO3 through binding to its LxxIxE motif. When this motif is mutated (FOXO3 2A), the dephosphorylation of FOXO3 T32 and S253 and nuclear translocation are prevented. The engraftment of the SERTA WT domain onto FOXO3 2A allowed efficient recruitment of PP2A-B55α. In contrast, no binding of PP2A-B55α to FOXO3 2A fused to the mutated SERTA domain could be detected (Fig. 2D). Strikingly, we observed efficient dephosphorylation of FOXO3 T32 and S253 when we engrafted SERTA WT, and this resulted in nuclear translocation of FOXO3 as predicted (Fig. 2, D to F). We conclude that α-helical recruitment modules can serve as substrate specifying elements for PP2A-B55.

### An engineered B55 inhibitor blocks binding of helical elements

Our data revealed that the conserved surface on B55α engages numerous substrates. On the basis of this, we predicted that a high-affinity peptide binding to this surface on B55α would inhibit PP2A-B55α through competitive substrate displacement. The generation of such an inhibitory peptide would further support our model of how PP2A-B55 recognizes substrates.

**Fig. 3.**
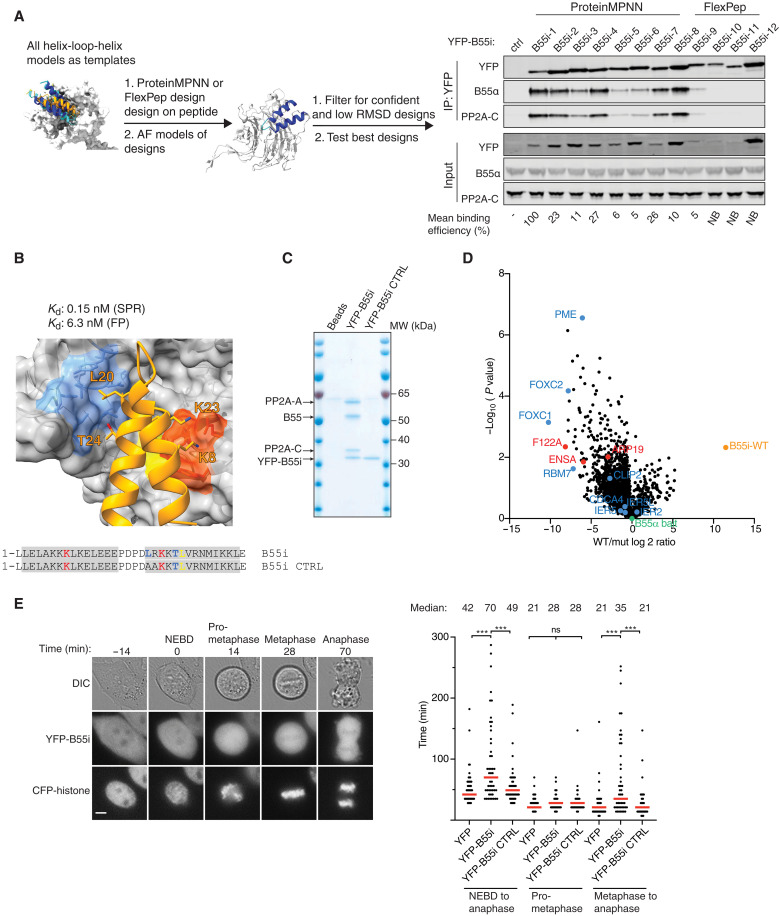
Generation of a specific B55 inhibitor. (**A**) Design pipeline for generating a specific and tight binder of B55 and test of the top designs by immunopurification. (**B**) Model of the B55i bound to B55 and measured *K*_d_ and *K*_i_ indicated above. (**C**) Coomassie stained gel of B55i and B55i CTRL purified from HeLa cells. MW, molecular weight. (**D**) B55 was affinity purified after incubation with either B55i or B55i CTRL peptides, and samples were analyzed by MS. (**E**) Left: Mitotic duration in cells expressing B55i measured by time-lapse microscopy. Scale bar, 5 μM. Right: Quantification of (E). Shown are pooled data from three independent experiments. Each circle represents the timing of a single cell. Red line indicates the median time. A Mann-Whitney *U* test was applied. ns, not significant. ***P < 0.001.

To generate this peptide, we turned to protein engineering using ProteinMPNN (*25*) and Rosetta FlexPepDesign (*37*) to generate a panel of artificial designs predicted to bind to PP2A-B55α as helix-loop-helix motifs (Fig. 3A, fig. S14, and table S4). A screen for binding to PP2A-B55α in cells by affinity purifying YFP-tagged versions of the peptides revealed that all peptides designed with ProteinMPNN bound, while only one of the FPD designs bound (Fig. 3A). The strongest binder stood out by the highest pLDDT and ProteinMPNN scores, the largest buried polar surface area, and also resulted in the smallest root mean square deviation (RMSD) to the starting structure upon refolding with AF2 (a measure used to estimate whether the design indeed will adapt the structure it was designed to adapt; fig. S14, A to E, and table S4). No clear correlation was observed for other measures calculated with the Rosetta Interface Analyzer protocol (*38*). We moved forward with the two strongest binders and measured their B55α binding affinity by surface plasmon resonance (SPR) and compared this to corresponding B55i CTRLs, in which residues crucial for the interaction were mutated. This revealed extremely tight binding of the inhibitors. In particular, inhibitor 1, termed B55i, showed strong B55α binding with an estimated *K*_d_ (dissociation constant) of 100 pM due to a low *k*_off_ rate (Fig. 3B and fig. S14F). This strong binding was supported by fluorescent polarization assays that measured a *K*_d_ of 6 nM (fig. S14G). The tight binding of B55i is achieved through several positively charged residues contacting negative patches on B55α and good packing of the hydrophobic core. Despite considerable efforts in both recombinant production and peptide synthesis, we have been unable to compare these affinity measurements to the helix-loop-helix binding elements identified in CDCA4, IER2, and AMOTL2. This is probably due to the highly insoluble nature of these protein elements. However, we did manage to produce several of the single helix type of B55 binders by protein expression and peptide synthesis and measured binding of full-length PME1, full-length Arpp19, and RBM7 129 to 158 to B55α in the low micromolar affinity range using the same SPR approach as for B55i (fig. S15). To test the specificity of B55i in cells, we affinity purified YFP-tagged versions of B55i and B55i CTRL and analyzed samples by colloidal staining and MS (Fig. 3C, fig. S14H, and table S3). This revealed a strong and very specific enrichment of all PP2A-B55 isoforms by B55i that was entirely abrogated by the mutations. These experiments emphasize the strong affinity and high selectivity of the designed inhibitor peptide for PP2A-B55 complexes.

To test the ability of the inhibitor to displace substrates from PP2A-B55α, we affinity purified B55α from cells in the presence of either B55i or B55i CTRL peptides and analyzed complexes by MS. This revealed that B55i specifically bound the complex and reduced the binding of several interactors containing a B55α binding site and the endogenous B55α inhibitors Arpp19, ENSA, and FAM122A (Fig. 3D and table S3). To test if B55i could block the biological function of PP2A-B55, we expressed YFP-tagged versions of B55i and B55i CTRL in HeLa cells and analyzed their effect on mitotic exit, which is a cellular process that depends on PP2A-B55 activity (*39*, *40*). Using live-cell time-lapse microscopy, we observed a significant delay in the metaphase to anaphase transition in cells expressing B55i inhibitor, while the duration of prometaphase was not affected (Fig. 3E). Thus, B55i causes in vivo phenotypes in line with the known function of PP2A-B55.

In conclusion, we used de novo protein design to generate a highly potent and specific PP2A-B55 inhibitor providing an important tool for dissecting and manipulating PP2A-B55 regulated signaling. Our ability to generate this inhibitor further supports our model of substrate recognition by this phosphatase.

### PP2A-B55 regulates NEXT complex function by binding a helical domain of RBM7

**Fig. 4.**
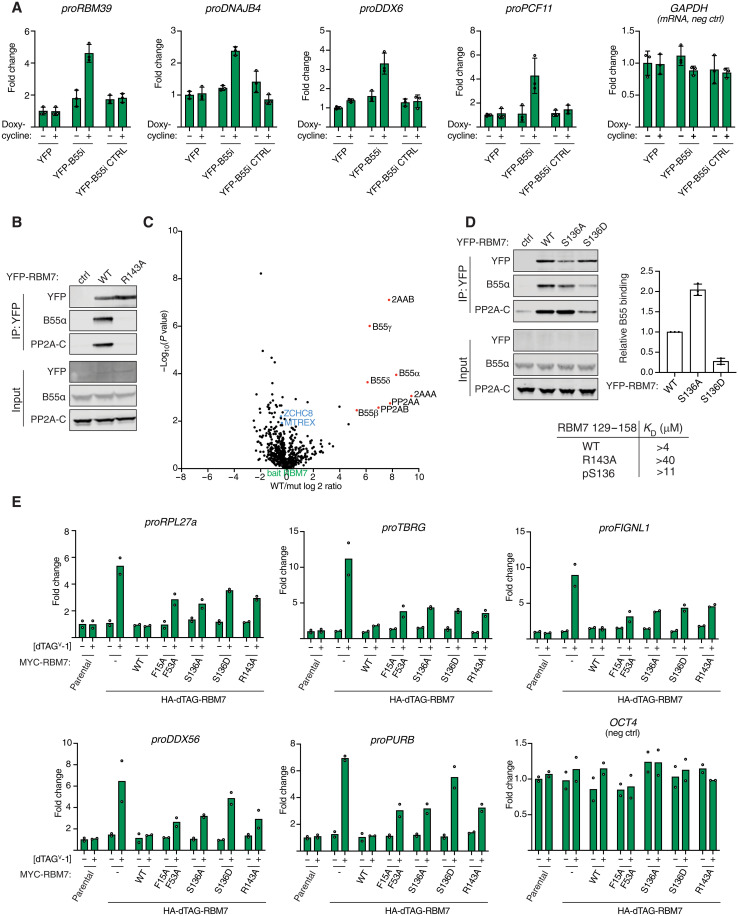
PP2A-B55 regulates NEXT complex function through binding RBM7. (**A**) Stable HeLa cell lines expressing the indicated constructs and RNA levels of indicated NEXT substrates measured by reverse transcription quantitative polymerase chain reaction (RT-qPCR). Quantifications of data from three biological replicates. Error bars show mean and SD. (**B**) IP of RBM7 constructs and monitoring binding to B55. (**C**) MS comparison of RBM7 WT and R143A with NEXT components highlighted in blue. (**D**) Binding of RBM7 S136A and S136D to PP2A-B55 and table of affinities measured of RBM7 peptides measured by SPR. Quantifications of data from three biological replicates. Error bars show mean and SD. (**E**) Endogenous RBM7 was tagged with dTAG, allowing the rapid removal of RBM7; cells were complemented with the indicated RBM7 variants; and the indicated RNAs were quantified by RT-qPCR. Quantifications of data from two biological replicates and the average are shown.

Our characterization of PP2A-B55α binding elements allowed us to address uncharacterized functions of this phosphatase. One of the helical binding elements that we mapped is located in the RBM7 protein that forms part of the nuclear exosome targeting (NEXT) complex. NEXT is composed of RBM7, ZCCHC8, and MTR4 (MTREX) and acts as an adaptor by channeling non-polyadenylated RNA to the ribonucleolytic RNA exosome complex for degradation (*41*, *42*). The phosphorylation of RBM7 in DNA damage conditions has previously been shown to impair normal function of the NEXT complex in RNA decay (*43*–*45*), suggesting a regulatory control mechanism mediated by phosphorylation/dephosphorylation. To explore a possible role of PP2A-B55α in regulating NEXT complex function, we induced the expression of B55i, or its mutant variant, in HeLa cells and monitored the levels of NEXT complex RNA substrates. This revealed a specific accumulation of the NEXT targets *proRBM39*, *proDNAJB4*, and *proDDX6* when B55i, but not the control, was induced, supporting an impact of PP2A-B55α on NEXT complex function (Fig. 4A and fig. S16).

To explore this in more detail, we turned to our AF2 modeling of the RBM7-B55α complex. This revealed a critical role of RBM7 R143 in binding to B55α, which we confirmed by immunopurifications of YFP-tagged full-length RBM7 in cells and by affinity measurements of RBM7 peptides spanning residues 129 to 158 (Fig. 4B, fig. S16, and see Materials and Methods for peptide sequences). Comparative MS analysis of immunoprecipitations of RBM7 wild type (WT) and the corresponding R143A variant detected no defect in NEXT complex assembly (ZCCHC8 and MTR4/MTREX) but demonstrated the efficient uncoupling of PP2A-B55 binding of RBM7 R143A (Fig. 4C). Moreover, our AF2 model showed that phosphorylation of S136 in RBM7, a reported functional phosphorylation site (*44*), would negatively regulate PP2A-B55 binding through electrostatic repulsion with acidic residues in B55. We confirmed this in binding assays using a phosphorylated RBM7 peptide or in cells using RBM7 S136D to mimic phosphorylation, suggesting a mechanism for how PP2A-B55 interaction with helical binding elements can be regulated (Fig. 4D and fig. S16).

To determine whether PP2A-B55 binding to RBM7 was required for in vivo function of the NEXT complex, we used a genetic complementation system in mouse embryonic stem (mES) cells where endogenous RBM7 could be inducibly degraded using the dTAG system (*46*), which allowed for the functional investigation of stably expressed RBM7 variants (Fig. 4E and fig. S16). As a functional readout, we monitored levels of a panel of NEXT RNA substrates in RBM7 WT, S136A/D, and R143A complemented cells. As controls, we included the RBM7 F15A/F53A variant with diagnostic point mutations in the RNA recognition motif domain (*47*) and also measured RNA levels of *OCT4* mRNA, which is not a NEXT substrate. While RBM7 WT fully suppressed the increased NEXT substrate levels, arising upon removal of endogenous RBM7, both the RBM7 S136 and R143 mutants failed to do so (Fig. 4E). Thus, regulated binding of PP2A-B55 to RBM7 appears important for NEXT function. We anticipate that phosphorylation sites in RBM7 itself and sites in NEXT complex-associated factors are dephosphorylated by PP2A-B55 to regulate RNA turnover in response to cellular cues. This is consistent with our observation of changes in RBM7 phosphorylation patterns when uncoupled from PP2A-B55 (fig. S5) and recent phosphoproteomic studies identifying several phosphosites on ZCCHC8 to be targets of PP2A-B55 (*29*, *48*).

## DISCUSSION

Here, we uncover how PP2A-B55 binds to cellular binding partners to regulate signaling pathways. A general concept emerging from our work is that PP2A-B55 binds to α helices in proteins. Despite limited sequence identity, these helices engage many of the same hydrophobic and charged regions on B55α. This mechanism of binding is distinct from that of SLiMs, representing a distinct mechanism by which PP2A-like phosphatases bind substrates. Although p107 was originally proposed to bind to PP2A-B55 via a SLiM, recent work argues that this SLiM is located in a helical element binding to the surface of B55 that we have described here (*23*). We observe a high degree of flexibility in the composition of binding α helices. This may allow for them to arise de novo through a limited number of mutations similar to how SLiMs can arise de novo. Thus, during evolution, PP2A-B55 can readily be integrated into signaling programs to tune their outputs and dynamics. We cannot exclude that alternative binding mechanisms for binding to PP2A-B55 exists, which could involve other structural elements or motifs in disordered regions. Our AF2 models of seven instances suggest that this is indeed the case, but additional future work is needed to investigate this. Our work argues that several of the α helices we identified act as substrate specifiers, a claim that is strongly supported by our ability to turn FOXO3 into a PP2A-B55 substrate by fusing it to the SERTA domain from CDCA4. We anticipate that once bound, PP2A-B55 can dephosphorylate several proteins in the vicinity of its binding site. Recent cryo-EM structures of endogenous inhibitors revealed that these also bind this conserved surface of B55 using short α helices while making additional contacts to the catalytic subunit (*22*). Thus, the function of inhibitors is twofold in blocking both substrate recruitment and activity.

Inspired by how the endogenous PP2A-B55 inhibitors work, we used deep learning protein design to generate a potent and highly specific B55 inhibitor that can outcompete substrate binding. We show that this inhibitor can be used to dissect PP2A-B55 functions in biological pathways such as cell division and RNA degradation. This design further reinforces the importance of the B55 binding site for biological function and regulation. Thus, our work outlines a general strategy for how de novo protein design can generate powerful tools for dissecting biological questions and provide tools for therapeutic proof of principle studies. An alternative strategy to our approach is to start from knowledge of the binding site only, as successfully reported for recent deep learning–based designs (*49*).

Our work highlights how the ability of AF2 to model protein-protein interactions at large scale can be used to obtain insight into biological systems. Other examples of the use of deep learning structure predictions in this space include work on host-pathogen interactions (*50*, *51*).

Collectively, we provide important insight into how PP2A-B55 achieves binding specificity and a foundation for understanding and precisely engineering PP2A-B55 programs to dissect cellular signaling throughout the eukaryotic domain of life.

## MATERIALS AND METHODS

### Expression constructs and immunoprecipitation

Standard cloning techniques were used throughout. Point mutations were introduced by whole plasmid polymerase chain reaction (PCR). All constructs were fully sequenced. Synthetic DNA was purchased from GeneArt, Life Technologies and cloned into pcDNA5/FRT/TO (Invitrogen) expression vector containing YFP, resulting in the indicated YFP fusion proteins. These constructs were transiently transfected into HeLa cells 24 hours before harvesting cells. Cells were lysed in lysis buffer [50 mM tris-HCl (pH 7.5), 50 mM NaCl, 1 mM EDTA, 1 mM DDT, and 0.1% NP-40]. Complexes were immunoprecipitated at 4°C in lysis buffer with green fluorescent protein (GFP)–Trap (ChromoTek) and washed in lysis buffer. Precipitated protein complexes were washed three times in lysis buffer, eluted in 2× SDS sample buffer, and subjected to Western blotting or MS as indicated.

### B55 inhibitor peptide competition for MS analysis

Ten full 15-cm^3^ dishes with HeLa FRT cells stably expressing Venus-B55 alpha were induced with doxycycline (10 ng/ml) for 24 hours and collected by trypsinization. Pellets from one dish were lysed in 400 μl of lysis buffer [100 mM NaCl, 50 mM tris (pH 7.4), 0.1% NP-40, and 1 mM DTT supplemented with protease and phosphatase inhibitors (Roche)]. Lysate was sonicated for 10 cycles (30-s on, 30-s off) at 4°C and kept on ice for 20 min and cleared for 45 min 4°C 20,000*g*. Next, the lysate was pooled, and 200 μl of pre-equilibrated GFP-trap beads were added, mixed, and divided into 10 Lo-bind tubes (Eppendorf). Either WT or ctrl/mutant inhibitor was added to a final concentration of 2 μM. Immunoprecipitations were incubated for 1 hour at 4°C, washed three times with 1 ml of lysis buffer, and eluted in 40 μl 2× Laemmli sample buffer.

### Computational methods

#### Screen of binders

Predictions with AF2 multimer v2.3 were run between the 256 proteins of the pull-down with the following parameters using A100 and A30 GPU-s:

{

“num_queries”: 1,

“use_templates”: false,

“num_relax”: 0,

“msa_mode”: “mmseqs2_uniref_env”,

“model_type”: “alphafold2_multimer_v3”,

“num_models”: 5,

“num_recycles”: null,

“recycle_early_stop_tolerance”: null,

“num_ensemble”: 1,

“model_order”: [ 1,2,3,4,5],

“keep_existing_results”: true,

“rank_by”: “multimer”,

“max_seq”: 508,

“max_extra_seq”: 2048,

“pair_mode”: “unpaired_paired”,

“pairing_strategy”: “greedy”,

“host_url”: “ https://api.colabfold.com “,

“user_agent”: “colabfold/1.5.2 (4991d3ee56dce5e7214709dd84784bb6749b2544)”,

“stop_at_score”: 100,

“random_seed”: 0,

“num_seeds”: 1,

“recompile_padding”: 10,

“commit”: “4991d3ee56dce5e7214709dd84784bb6749b2544”,

“use_dropout”: false,

“use_cluster_profile”: true,

“use_fuse”: true,

“use_bfloat16”: true,

“version”: “1.5.2”

}

The resulting complexes were filtered by calculating the interface residues between the conserved binding site residues of B55 and the partner (see fig. S1B and table S1). To speed up computation, we first calculated the distance between all Cβ atoms of the two chains and selected those within 8 Å; then for these residues, we measured distances between all atoms of the two chains and used the cutoff of 4 Å to define interacting residues. On the basis of our observation that pLDDT confidence of flanking regions around otherwise well-predicted regions can drag down the average pLDDT calculated in the next step, we discarded residues with pLDDT below 50 from further calculations. For the remaining interfaces, we calculated the following metrics if more than seven interface residues remained, otherwise we discarded the model:

1) The average pLDDT for the binding partner of B55 over its interface residues selected previously.

2) Median iPAE (interface PAE) by selecting the values defined by the peptide (rows) and receptor (columns) interface residues from the PAE matrix and calculating their median.

For models that did not meet these criteria, we set the average pLDDT to 0 and iPAE to 30.

We then selected the complexes with iPAE < 10 and average interface pLDDT > 70. Last, the complexes were manually inspected.

We note that the recently published solved structures of B55 bound to ARPP19 and FAM122A were not in the set used to train AF2.

#### Computational alanine scanning

Computational alanine scanning was performed with a local installation of Robetta alanine scanning (*52*) on the relaxed structures (table S2). Residues with predicted δδG > 1 kcal/mol were predicted to be interface hotspots.

#### Design process for de novo binders

The structures of AMOTL2, IER2, CDCA4, and Zds1 were submitted to design with ProteinMPNN (*25*) using the interface at HuggingFace (https://huggingface.co/spaces/simonduerr/ProteinMPNN). One hundred sequences were generated for each input template with the default model, temperature of 0.1, and backbone noise of 0.3.The chain of B55 was fixed during design, and the binder chain was fully designable. The resulting sequences were refolded with AF2 using local runs with default parameters. The average pLDDT of the binder residues was calculated, as well as the RMSD between the template and the refolded designed binders. Designs with average pLDDT > 90 were manually inspected.

For FlexPepDesign, the receptor was first prepacked with the following command:

-s <af_structure>

-ex1

-ex2aro

-use_input_sc

-flexpep_prepack

-nstruct 1

-scorefile ppk.score.sc

-flexpep_score_only

-out:path:pdb input

-out:path:score output

-unboundrot input/b55_unbound.pdb

Then FlexPepDock with the following commands was run on the prepacked structure:

-ex1

-ex2aro

-use_input_sc

-nstruct 200

-flexpep_score_only

-scorefile design.sc

-unboundrot input/b55_unbound.pdb

-out:file:silent_struct_type binary

-overwrite

-flexPepDocking:pep_refine

-flexPepDocking:design_peptide

-s <prepack_structure>

-out:path:pdb output/

-resfile input/resfile

With the following resfile:

NATRO

START

* C ALLAA xc

Then, refolding was done using AF2 multimer v2, as described previously.

#### Visualization of structural models

Structural models were visualized using ChimeraX (*53*). The conservation plot of B55 was generated using ConSurf (*54*).

#### Live-cell analysis

Live-cell analysis was performed on a Deltavision Elite system using a ×40 oil objective with a numerical aperture of 1.35 (GE Healthcare). The DeltaVision Elite microscope was equipped with a CoolSNAP HQ2 camera (Photometrics). Cells were seeded in eight-well Ibidi dishes (Ibidi), and before filming, the medium was changed to Leibovitz’s L-15 (Life Technologies). Appropriate channels were recorded for the times indicated. For transient transfections, DNA constructs were transfected into HeLa cells using jetOPTIMUS reagent (Polyplus) 24 hours before analysis. The nuclear/cytoplasmic distribution of YFP-FoxO3 was analyzed using SoftWoRx (GE Healthcare) software.

#### Cell culture

HeLa cells were maintained in Dulbecco’s modified Eagle’s medium (DMEM) GlutaMAX containing penicillin (100 U/ml), streptomycin (100 mg/ml), and 10% fetal calf serum (all from Thermo Fisher Scientific). Stable HeLa cell lines were generated using the T-Rex doxycycline inducible Flp-In system (Invitrogen) and cultivated similar to HeLa cells with the addition of blasticidin (BSD) (5 mg/ml) and hygromycin B (100 mg/ml). *Escherichia coli* DH5alpha were maintained and propagated using standard microbiological procedures. The following drug concentrations were used: 2.5 mM thymidine and doxycycline (10 ng/ml) unless otherwise stated.

#### Expression and purification of proteins

Β55α was cloned into pCPR0197, allowing expression in HEK293 cells as a His-Strep-TEV fusion protein. Following harvesting, the cell pellet was resuspended in lysis buffer [100 mM tris (pH 8.0), 150 mM NaCl, 1 mM EDTA, tris(2-caboxyethyl)phosphine (TCEP), protease inhibitors, and benzonase] and cells lysed by sonication. Following clarification by centrifugation and filtration, the lysate was loaded on a Strep affinity column and washed with buffer A [100 mM tris (pH 8.0), 150 mM NaCl, 1 mM EDTA, and TCEP] and eluted with buffer A + 2.5 mM desthiobiotin. The peak fractions were pooled and diluted with 100 mM tris (pH 8.0) and loaded onto a MonoQ column. The column was washed with buffer M [100 mM tris (pH 8.0), 50 mM NaCl, 10% glycerol, and TCEP] and eluted with a 50 mM-1 to 1 M NaCl gradient in buffer M. Individual fractions were frozen.

His-PME1 was expressed in BL21(DE3) at 18°C overnight. Cell pellets were resuspended in buffer L [50 mM NaP (pH 7.5), 300 mM NaCl, 10 mM imidazole, 10% glycerol, 0.5 mM TCEP, and protease inhibitors] and lysed by sonication followed by centrifugation to clarify lysate. The lysate was loaded on a His affinity column and washed with buffer W [50 mM NaP (pH 7.5), 300 mM NaCl, 30 mM imidazole, 10% glycerol, and 0.5 mM TCEP] and eluted with the same buffer but containing 500 mM imidazole. Peak fractions were pooled, concentrated, and run on a Superdex 200 26/60 equilibrated with buffer GF [50 mM NaP, 150 mM NaCl, 10% glycerol, and 0.5 mM TCEP], and peak fractions were pooled.

#### Surface plasmon resonance

All SPR experiments were performed at 25°C on a Biacore T200 instrument equipped with CM5 sensor chips (Cytiva, Uppsala, Sweden). SPR running buffers and amine-coupling reagents [*N*-ethly-*N*′-(3-dimethlyaminoproply)carbodiimide, *N*-hydroxysuccinimide, and ethanolamine HCl] were purchased from Cytiva. The Twin-Strep-Tag Capture Kit was from IBA Lifesciences (IBA GmbH, Germany). Peptides were purchased from Peptide 2.0 Inc. (Chantilly, USA). A 1× PBS-P [11.9 mM NaH_2_PO4-Na_2_HPO4 (pH 7.4), 137 mM NaCl, 2.7 mM KCl, and 0.005% (v/v) surfactant P20] was used as running buffer for Strep-TactinXT immobilization onto flow cells 1 and 2 (Fc1 and Fc2) of the CM5 SPR sensor chip following the protocol from the manufacturer. Subsequently, Twin-Strep–tagged B55α at 20 nM was captured on the Fc2 channel of the Strep-TactinXT–coated chip. The Fc1 remained unmodified and was used as reference for subtraction of systematic instrumental drift. Approximately 550 relative units of B55α were obtained with a flow rate of 5 μl/min and a contact time of 100 s. The buffer used for B55α capture and the following SPR binding experiments was 20 mM tris (pH 8.0), 250 mM NaCl, 0.5 mM TCEP, and 0.05% Tween 20. Peptide and full-length proteins (PPME1, Arpp19 *S62E*) threefold serial dilutions were prepared in the SPR running buffer from the primary stocks, and a series of concentrations (two or three) run in duplicates. Peptides and full-length proteins (PPME1, Arpp19 *S62E*) were injected sequentially over the two flow cells at a flow rate of 30 or 60 μl/min (B55i) for 80 s. The dissociation rate of the complexes was monitored for 480 or 600 s (B55i-1/B55α). All binding experiments were run at least in duplicates to confirm the reproducibility of the assay. Data processing and fitting were done using the BiaEvaluation software (v. 3.2.1, Cytiva, Uppsala, Sweden). The raw sensorgrams were double referenced (referring to the subtraction of the data over the reference surface and the average of the buffer injections from the binding responses). The equilibrium *K*_d_ were determined by plotting the equilibrium responses levels (Req) against the analyte concentrations and fitting to a steady-state model. For the Cdca4-1/B55α complex, the association and dissociation phases over all replicates were globally fit using a 1:1 interaction model yielding single values for the *k*_a_ and the *k*_d_. The equilibrium dissociation constant, *K*_d_, is the rate of the *k*_d_ over the *k*_a_. We used the following B55i and RBM7 peptides for SPR measurements: B55i-1 wt: LLELAKKKLKELEEEPDPDLRKKTLVRNMIKKLEW, B55i-1 ctrl: LLELAKKKLKELEEEPDPDAAKKTLVRNMIKKLEW, B55i-2 wt: EEVERLLKLAEEKLKDESLSLTKVLLRNLIESIW, B55i-2 ctrl: EEVERLLKLAEEKLKDESLSLTKKALLRNAIESIW, RBM7 wt: QIIQRSFSSPENFQRQAVMNSALRQMSYGGW, RBM7 R143A: QIIQRSFSSPENFQAQAVMNSALRQMSYGGW, and RBM7 pS136: QIIQRSF(Sp)SPENFQAQAVMNSALRQMSYGGW.

#### Fluorescent polarization assay

The binding affinity between the fluorescent peptide probe (FITC-PEG2-LLELAKKKLKELEEEPDPDLRKKTLVRN-Nle-IKKLEW) and B55α was determined as the *K*_d_ value by saturation binding experiments, where increasing concentrations of B55α (1.8 to 500 nM) were added to a fixed concentration of peptide probe (3 nM). The assay was performed in a 1× HBS-T buffer [10 mM Hepes, 150 mM NaCl, and 0.005% Tween 20 (pH 7.4)] using black flat-bottom 384-well plates (Corning Life Sciences, NY) and a volume of 30 μl per well. The assay plate was spun down to ascertain proper mixing and the removal of potential air bubbles before measuring the fluorescent polarisation (FP) levels on a Safire2 plate reader (Tecan, Mannedorf, Switzerland). The *g* factor was adjusted at each experiment so that a series of three blank wells containing probe but no B55α defined the baseline FP value. The probe was measured at an excitation/emission value of 470:535 nm. The FP values were fitted to the one-site specific binding equation: *Y* = *B*_max_ × *X*/(*K*_d_ + *X*), with *B*_max_ being the maximal FP value, *X* the PP2A B55α concentration, and *Y* the variable FP values. The *K*_d_ values were derived from the resulting binding saturation curve as being equal to the B55α concentration, where the curve is half-saturated.

#### Mass spectrometry

Pull-downs were analyzed on a Q-Exactive Plus quadrupole or Fusion Orbitrap Lumos mass spectrometer (Thermo Fisher Scientific) equipped with Easy-nLC 1000 or 12,000 (Thermo Fisher Scientific) and nanospray source (Thermo Fisher Scientific). Peptides were resuspended in 5% methanol/1% formic acid and loaded onto a trap column [1 cm in length, 100 μm in inner diameter, ReproSil, C18 AQ 5-μm 120-Å pore (Dr. Maisch, Ammerbuch, Germany)] vented to waste via a micro-tee and eluted across a fritless analytical resolving column (35 cm in length, 100 μm in inner diameter, ReproSil, C18 AQ 3-μm 120-Å pore) pulled in-house (Sutter P-2000, Sutter Instruments, San Francisco, CA) with a 45-min gradient of 5 to 30% liquid chromatography–MS (LC-MS) buffer B (LC-MS buffer A: 0.0625% formic acid and 3% acetonitrile (ACN); LC-MS buffer B: 0.0625% formic acid, 95% ACN Q-Exactive Plus quadrupole or 0.0625% formic acid and 80% ACN Fusion Orbitrap Lumos mass).

Raw data were searched using COMET (*55*)(release version 2014.01) in high-resolution mode against a target-decoy (reversed) (*56*) version of the human proteome sequence database (UniProt; downloaded February 2020, 40704 entries of forward and reverse protein sequences) with a precursor mass tolerance of ±1 Da and a fragment ion mass tolerance of 0.02 Da, and requiring fully tryptic peptides (K, R; not preceding P) with up to three miscleavages or no enzyme for proteinase K digests. Static modifications included carbamidomethylcysteine, and variable modifications included oxidized methionine and STY phosphorylation. Searches were filtered using orthogonal measures including mass measurement accuracy (±3 parts per million), Xcorr for charges from +2 to +4, and dCn targeting a <1% false discovery rate at the peptide level. Quantification of LC-MS/MS spectra was performed using MassChroQ (*57*) and the iBAQ method (*58*). All data were analyzed using R 4.3.0. Missing values were imputed from a normal distribution. To be included in further analysis, proteins had to be identified with more than 1 total peptide and quantified in two or more replicates of each sample. In each dataset, the abundance of the protein of interest was normalized to be equal across all samples. Statistical analyses were done using a two-tailed Student’s *t* test.

#### mES cell culture and cell line generation

mES cell lines were descendants of the parental E14TG2a cell line (male genotype, XY). mES cells were cultured on 0.2% gelatin-coated plates in 2i/LIF containing medium (1:1 mix of DMEM/F12 (Givco) and Neurobasal (Gibco) supplemented with 1x Pen-Strep (Gibco), 2 μM Glutamax, 50 μM beta-mercaptoethanol (Gibco), 0.1 mM Non-Essential Amino Acids (Gibco), 1 mM sodium pyruvate (Gibco), 0.5x N2 Supplement (Gibco), 0.5x B27 Supplement (Gibco), 3 μM GSK3-inhibitor (CHIR99021), 1 μM MEK-inhibitor (PD0325901) and Leukemia Inhibitory Factor (LIF, produced in house) at 37°C, 5% CO_2_. Cells were passaged every 48–72 hours by aspirating medium, dissociating cells with 0.05% Trypsin-EDTA (Gibco) briefly at 37°C before neutralizing with an equal volume of 1x Trypsin Inhibitor (Sigma) and gentle disruption by pipetting. Cells were pelleted by centrifugation to remove Trypsin before resuspending in 2i/leukemia inhibitory factor (LIF) medium and plating ~8 × 10^4^ cells/ml. CRISPR-Cas9–mediated genomic knock-ins (KIs) of N-terminal 2xHA-FKBP-V(dTAG) tags were carried out using homology-dependent repair (HDR) donor vectors. HDR vectors were cloned to contain gene specific 5′ and 3′ homology arms (~500 base pairs) amplified from WT mES cell genomic DNA and cloned into pGNT vectors along with either HYG-P2A-2xHA-FKBP-V or PUR-P2A-2xHA-FKBP-V tagging cassettes. Single guide RNAs (sgRNAs) targeting the transcriptional start site of genomic loci were cloned into pSLCas(BB)-2A-GFP vectors (pX458; Addgene plasmid ID: 48138) as previously described (*59*). Cells were transfected using Lipofectamine 3000 (Thermo Fisher Scientific) with two donor plasmids harboring distinct selection markers (HYG/PUR) along with a sgRNA/Cas9 vector in a 1:1:1 ratio. Cells were maintained under double HYG/PUR selection to increase the likelihood of homozygous KI clones. Single-cell clones were expanded and screened by Western blotting analysis before confirming genomic integrations by Sanger sequencing of the target locus.

#### cDNA cloning and exogenous expression of RBM7

Mouse RBM7 cDNA constructs were cloned using a full-length mouse pUC[mRBM7] cDNA plasmid (Sino Biological, MG5399-U) as a template. RBM7 constructs were amplified by Phusion DNA polymerase (NEB) using standard conditions, and mutations were introduced in primer sequences. Fragments were cloned into piggyBAC (pB) vectors harboring a N-terminal MYC tag and BSD resistance selection marker using the NEBuilder HiFi DNA assembly. HA-dTAG-RBM7 cells were transfected with pB[MYC-RBM7^x^] BSD plasmids along with a pB transposase expressing vector (pBASE) using Viafect transfection reagent (Promega). Cell pools were selected using BSD for 7 to 10 days or until negative control cells no longer survived. The expression of constructs was validated by Western blotting analysis using MYC antibodies.

#### Western blotting

Whole-cell protein lysates were prepared using RSB100 lysis buffer [10 mM tris-HCl (pH 7.5), 100 mM NaCl, 2.5 mM MgCl_2_, 0.5% NP-40, and 0.5% Triton X-100] freshly supplemented with protease inhibitors (Roche). Samples were denatured by the addition of NuPAGE Loading buffer (Invitrogen) and NuPAGE Sample Reducing Agent (Invitrogen) before boiling at 95°C for 5 min. SDS–polyacrylamide gel electrophoresis was carried out on NuPAGE 4–12% Bis-Tris gels (Invitrogen). Western blotting analysis was carried out using standard protocols with the antibodies listed in Table 1 and horseradish peroxidase–conjugated secondary antibodies (Agilent). Bands were visualized by using SuperSignal West Fempto ECL substrate (Thermo Fisher Scientific) and captured using an Amersham ImageQuant 800 imaging system (GE Healthcare). Images were processed using ImageJ (v.1.53) (*60*). Examples of uncropped Western blots are provided in fig. S17.

**Table 1.** Antibodies used in this study.

Target	Host	Source
ACTIN	Mouse	Sigma-Aldrich (A2228)
ARS2	Rabbit	GeneTex (GTX119872)
HA	Rat	Sigma-Aldrich (11867423001)
MTR4	Rabbit	Abcam (ab70551)
MYC	Rabbit	Cell Signaling Technology (2278)
VINCULIN (VCL)	Mouse	Sigma-Aldrich (V9131)
ZCCHC8	Rabbit	Novus Biologicals (NB100-94995)
ZCCHC8	Mouse	Abcam (ab68739)
YFP	Rabbit	Generated in house
FOXO3 pS253	Rabbit	Moravian Biotechnology
FOXO3 pT32	Rabbit	Moravian Biotechnology
B55α	Mouse	Cell Signaling Technology (#5689S)
PP2A catalytic subunit	Mouse	Milipore (05-421)
PP2A scaffold subunit	Rabbit	Cell Signaling Technology (2041)

#### RNA isolation and RT-qPCR analysis

Total RNA was isolated using TRIzol (Invitrogen) according to the manufacturer’s instructions. RNA extracts were treated with TURBO DNase (Invitrogen) according to the manufacturer’s instructions, followed by cDNA synthesis from 2 μg of total RNA using SuperScript III reverse transcriptase (Invitrogen) and a mixture of 80 pmol of random primers (Invitrogen) and 20 pmol of oligo d(T)20VN (Merck). Quantitative PCR (qPCR) was performed using Platinum SYBR Green (Invitrogen) and an AriaMX Real-Time PCR machine (Agilent). Primers used for reverse transcription qPCR (RT-qPCR) are listed in Table 2.

**Table 2.** RT-qPCR primers used in this study. GAPDH, glyceraldehyde-3-phosphate dehydrogenase.

Target	Species	Forward	Reverse
proRPL27a	Mouse	CGTCGGAGTGCACTGTTCTT	GAAGTCTTGCCGATGCTCTG
proDDX56	Mouse	CCCTGACCCACAGAGTGACG	CCACAGAACCCTAATTCCTTTGCG
GAPDH	Mouse	TTGATGGCAACAATCTCCAC	CGTCCCGTAGACAAAATGGT
OCT4	Mouse	CAGCAGATCACTCACATCGCCA	GCCTCATACTCTTCTCGTTGGG
proTBRG	Mouse	GCCAGGTGTCGGGTAATTACAG	CGGTGTGCCGTTGTATACGG
proFIGNL1	Mouse	CTTGGCTTCCCGTTCACTGC	GTGCTCTCTACACTCCTGAGC
proPURB	Mouse	GACGCTCCCGGTTTCAGAGG	GGAGGTGATTGGCTCCACTG
proRBM39	Human	AATAGATTTCCCTGTCATTTGGAGC	TTTCCAAGGTTGTTTCAAAGCTCG
proDNAJB4	Human	TTTCTGGCGTTTCTGATTGA	ACCAAAACGCAGGTTGTTTA
proDDX6	Human	CACACCGACGAGAAAAGTTCG	CATTTCTCAATCACGTCGCGG
proPCF11	Human	CACAACAGCCACACCCAGCT	CCAACCTGGGAAGACAGCCC
GAPDH	Human	GTCTCCTCTGACTTCAACAGCG	ACCACCCTGTTGCTGTAGCCAA
RPLP0	Human	TGGTCATCCAGCAGGTGTTCGA	ACAGACACTGGCAACATTGCGG
