# Supplementary material for: Substrate recognition principles for the PP2A-B55 protein phosphatase
Source: Sci Adv. 2024 Oct 2;10(40):eadp5491. doi: 10.1126/sciadv.adp5491 (PMC11446282; doi:10.1126/sciadv.adp5491)
Supplement: Supplementary file 1 — Figs. S1 to S17 Table S1 Legends for tables S2 to S4 Legend for data S1 [file sciadv.adp5491_sm.pdf]

Science Advances
AAAS

Supplementary Materials for

Substrate recognition principles for the PP2A-B55 protein phosphatase

Thomas Kruse *et al.*

Corresponding author: Jakob Nilsson, jakob.nilsson@cpr.ku.dk

Sci. Adv. **10**, eadp5491 (2024)
DOI: 10.1126/sciadv.adp5491

The PDF file includes:

Figs. S1 to S17
Table S1
Legends for tables S2 to S4
Legend for data S1

Other Supplementary Material for this manuscript includes the following:

Tables S2 to S4
Data S1

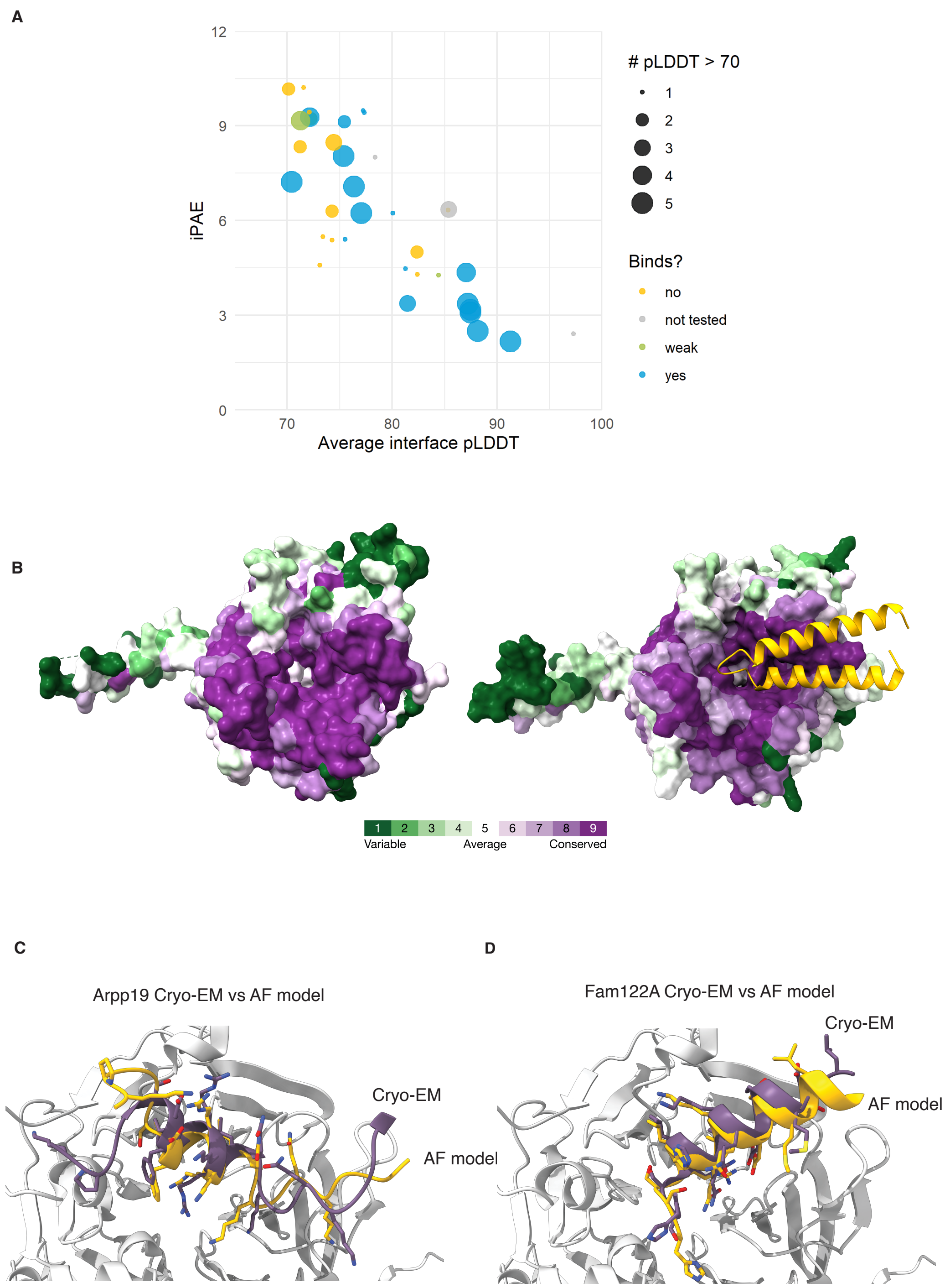

Supplemental Figure S1. AF score plots and comparison to cryo-EM models.
A) Plot showing iPAE and pLDDT score for the 36 models and whether they were binding to PP2A-B55α in immunoprecipitation experiments. The size of the dot indicates number of models with a pLDDT above 70. **B)** Surface conservation of B55α with CDCA4 modeled **C)** Overlay of the Arpp19 cryo-EM structure (PDB: 8TTB) with the AF2 model focusing on the helix binding B55. **D)** Overlay of the FAM122A cryo-EM structure (PDB: 8SO0) with the AF2 model focusing on the helix binding B55.

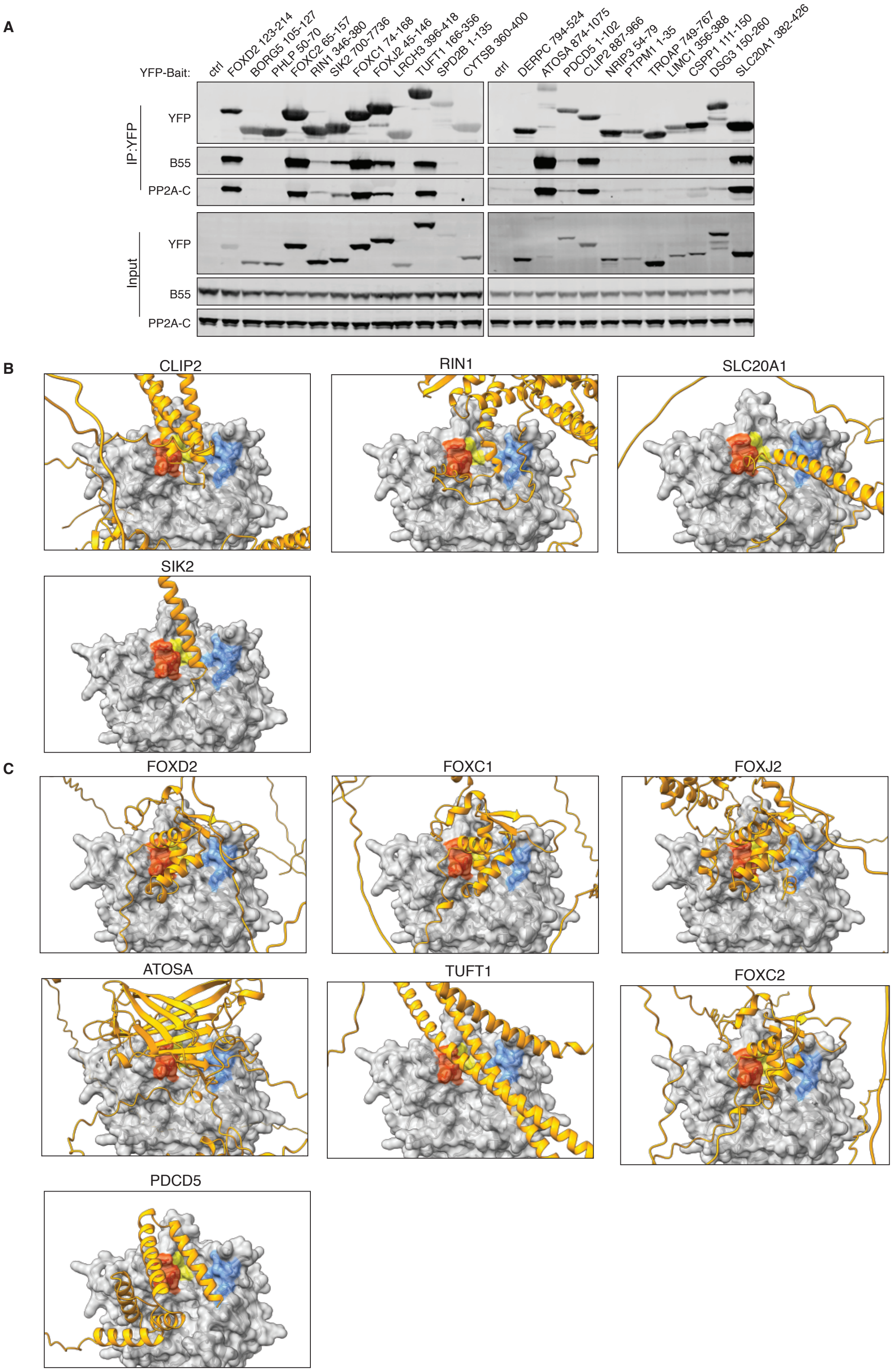

Supplemental Figure S2. Validation of novel binders and AF models of distinct binding mechanisms.

A) The indicated YFP tagged protein fragments were expressed in HeLa cells and following immunoprecipitation they were probed for binding to PP2A-B55α by western blot. B55 refers to B55α **B)** AF2 models of the indicated proteins binding to PP2A-B55 in a common manner. **C)** AF2 models of the indicated proteins binding to PP2A-B55 in a distinct manner.

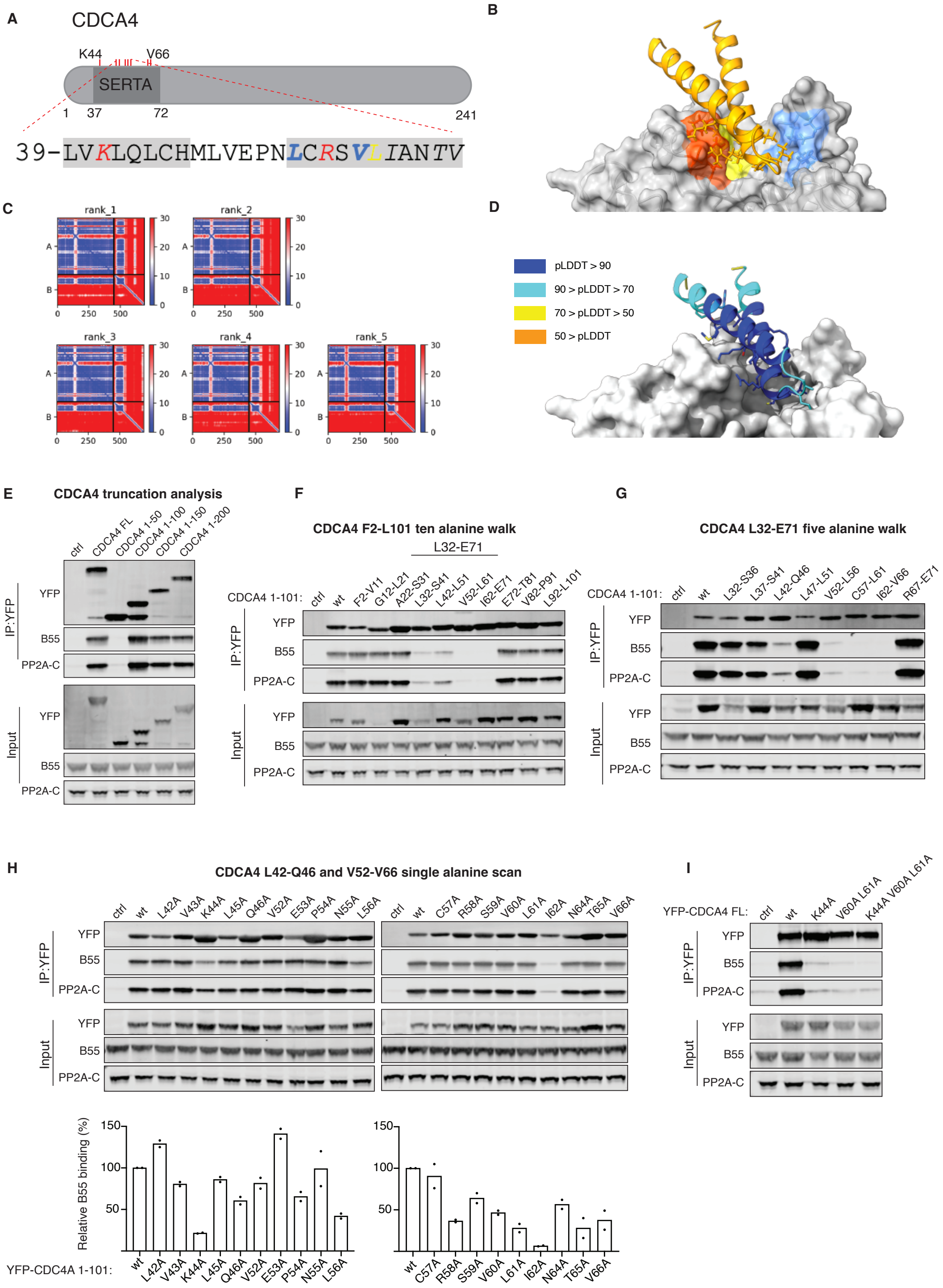

Supplemental Figure S3. CDCA4 data.
A) Schematic of CDCA4 and sequence binding PP2A-B55α. Amino acids crucial for binding are indicated by a red line in the schematic. **B**) AF2 model of CDCA4 bound to B55. **C**) Predicted aligned error (PAE) score for the 5 models. **D**) pLDDT score mapped onto model. **E-I**) The indicated YFP tagged protein fragments were expressed in HeLa cells and following immunoprecipitation they were probed for binding to PP2A-B55 by western blot. B55 refers to B55α. In H) the quantification of two biological replicates is shown with binding normalized to wild type protein. Bars visualize the average of the two biological replicates.

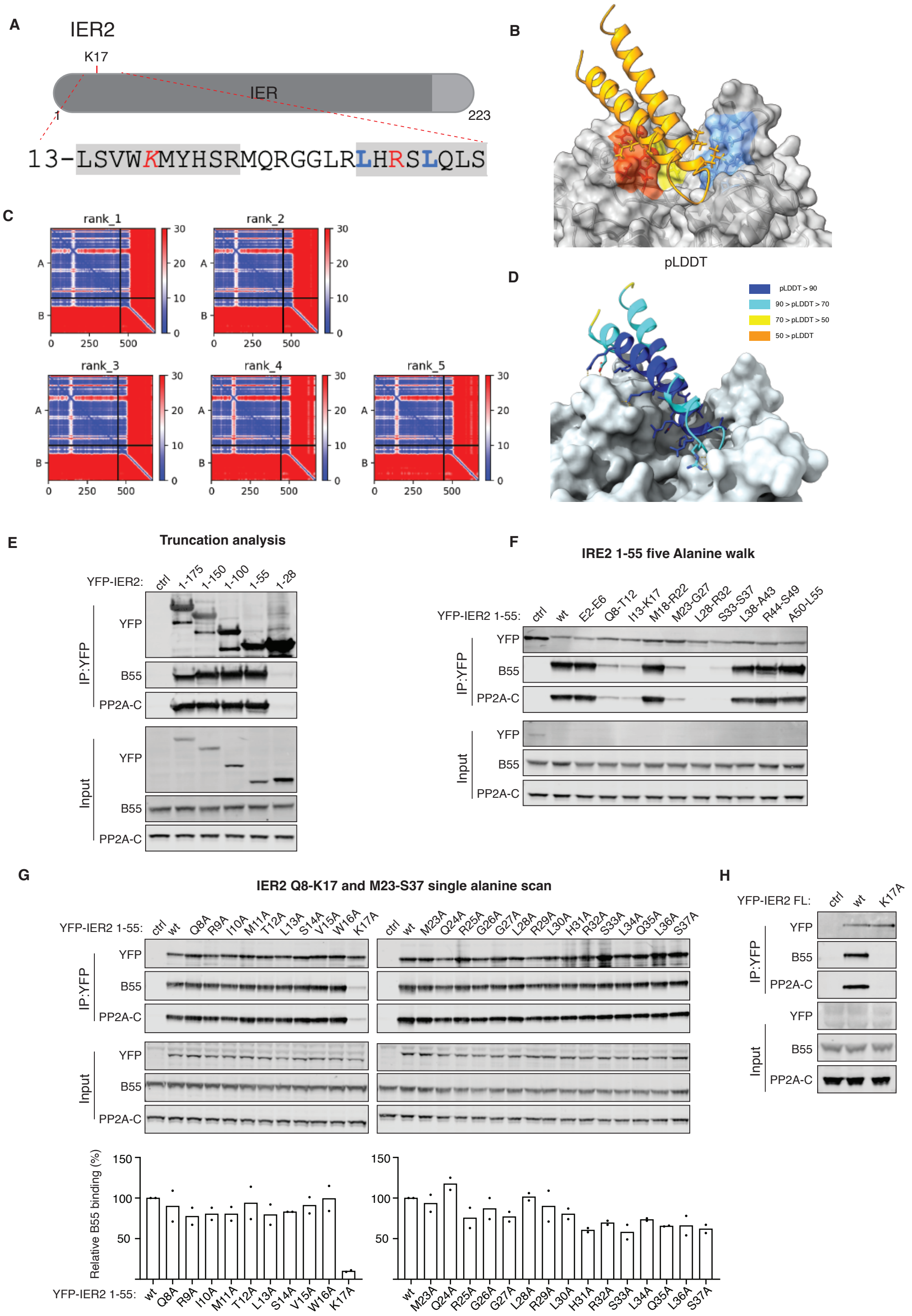

Supplemental Figure S4. IER2 data.

A) Schematic of IER2 and sequence binding PP2A-B55α. Amino acids crucial for binding are indicated by a red line in the schematic. **B**) AF2 model of IER2 bound to B55. **C**) Predicted aligned error (PAE) score for the 5 models. **D**) pLDDT score mapped onto model. **E-H**) The indicated YFP tagged protein fragments were expressed in HeLa cells and following immunoprecipitation they were probed for binding to PP2A-B55 by western blot. B55 refers to B55α. In G) the quantification of two biological replicates is shown with binding normalized to wild type protein. Bars visualize the average of the two biological replicates.

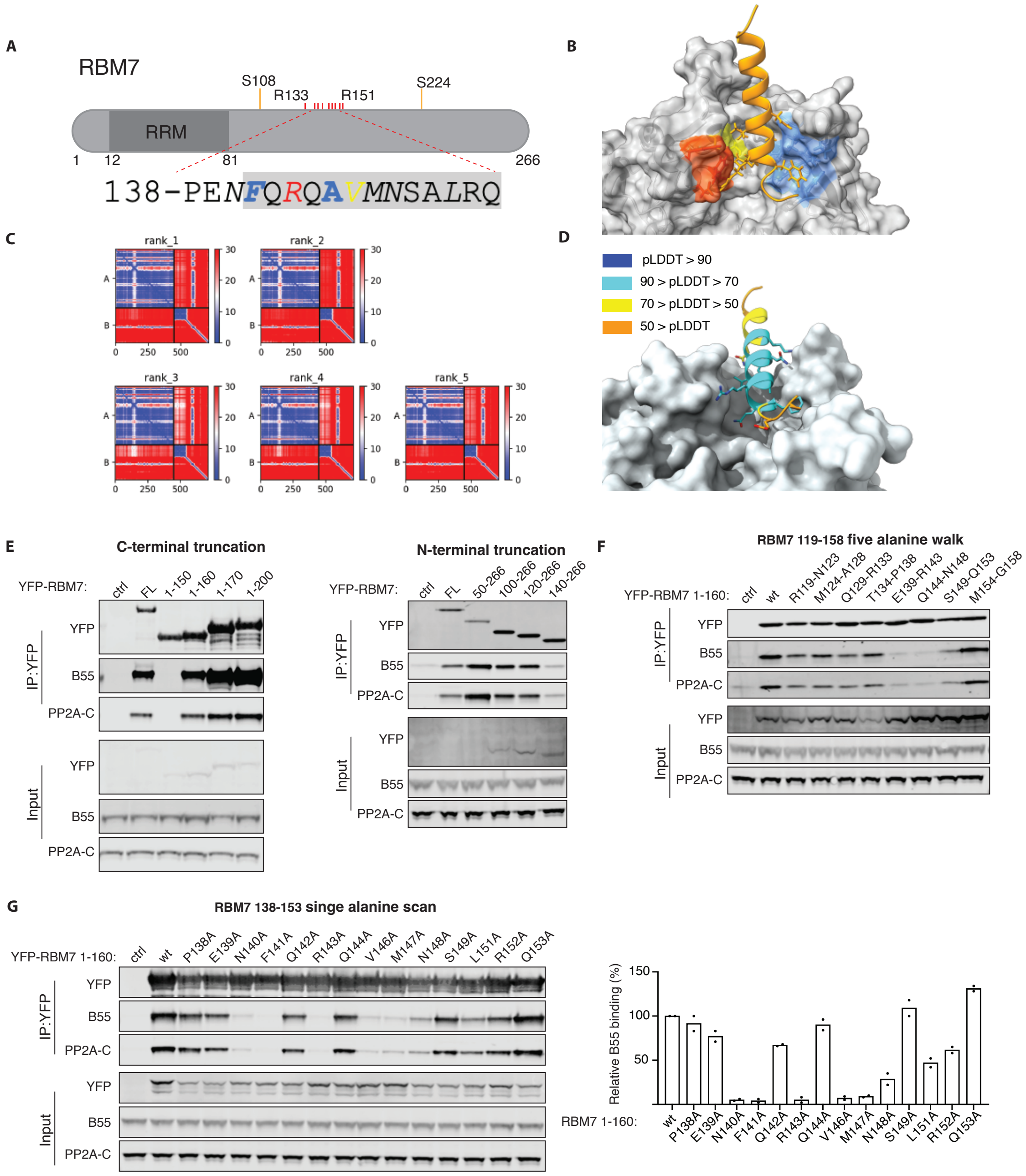

Supplemental Figure S5. RBM7 data.

A) Schematic of RBM7 and sequence binding PP2A-B55. Amino acids crucial for binding are indicated by a red line in the schematic. Phosphorylation sites with higher occupancy in RBM7 R143A indicated with yellow lines and amino acid. **B**) AF2 model of RBM7 bound to B55. **C**) Predicted aligned error (PAE) score for the 5 models. **D**) pLDDT score mapped onto model. **E-G**) The indicated YFP tagged protein fragments were expressed in HeLa cells and following immunoprecipitation they were probed for binding to PP2A-B55 by western blot. B55 refers to B55α. The quantification of two biological replicates is shown with binding normalized to wild type protein. Bars visualize the average of the two biological replicates.

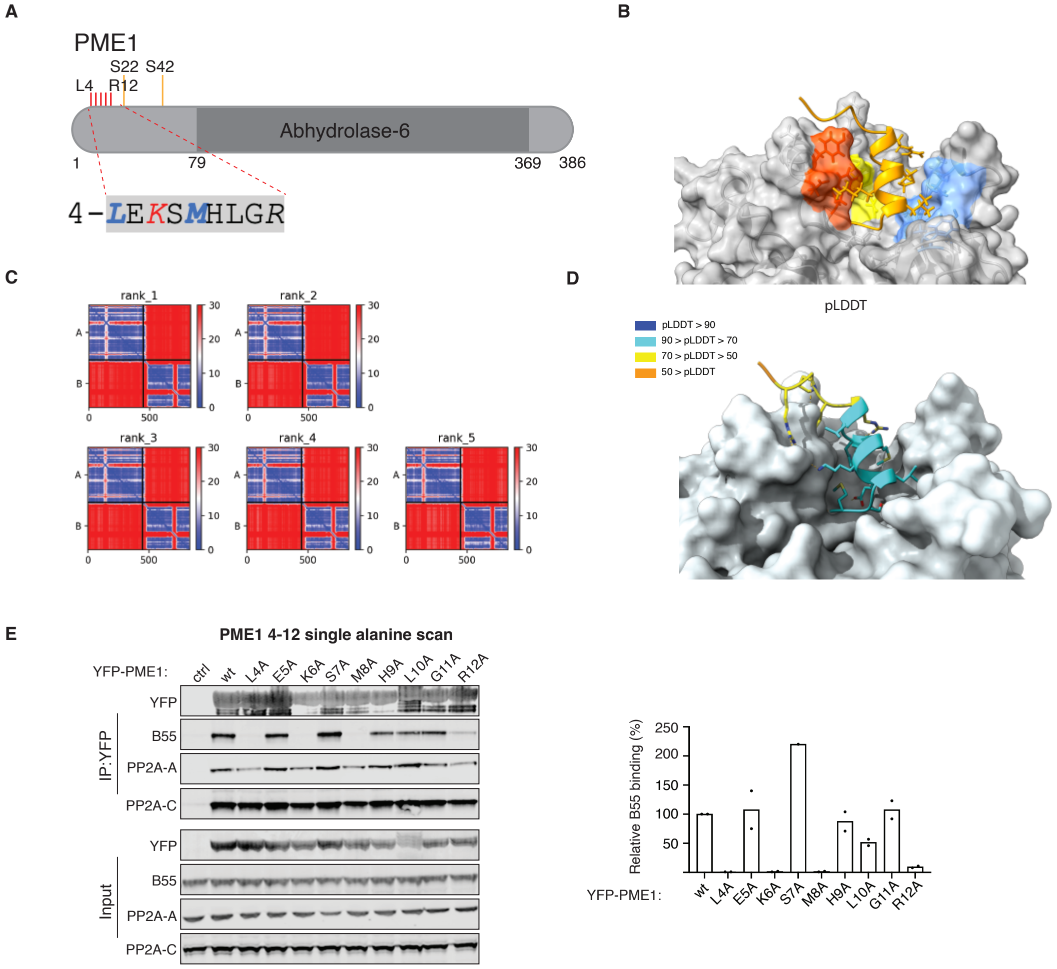

Supplemental Figure S6. PME1 data.

A) Schematic of PME1 and sequence binding PP2A-B55. Amino acids crucial for binding are indicated by a red line in the schematic. Phosphorylation sites with higher occupancy in PME1 L4A indicated with yellow lines and amino acid number. **B**) AF2 model of PME1 binding helix bound to B55. **C**) Predicted aligned error (PAE) score for the 5 models. **D**) pLDDT score mapped onto model. **E**) The indicated YFP tagged proteins were expressed in HeLa cells and following immunoprecipitation they were probed for binding to PP2A-B55 by western blot. B55 refers to B55α. The quantification of two biological replicates is shown with binding normalized to wild type protein. Bars visualize the average of the two biological replicates.

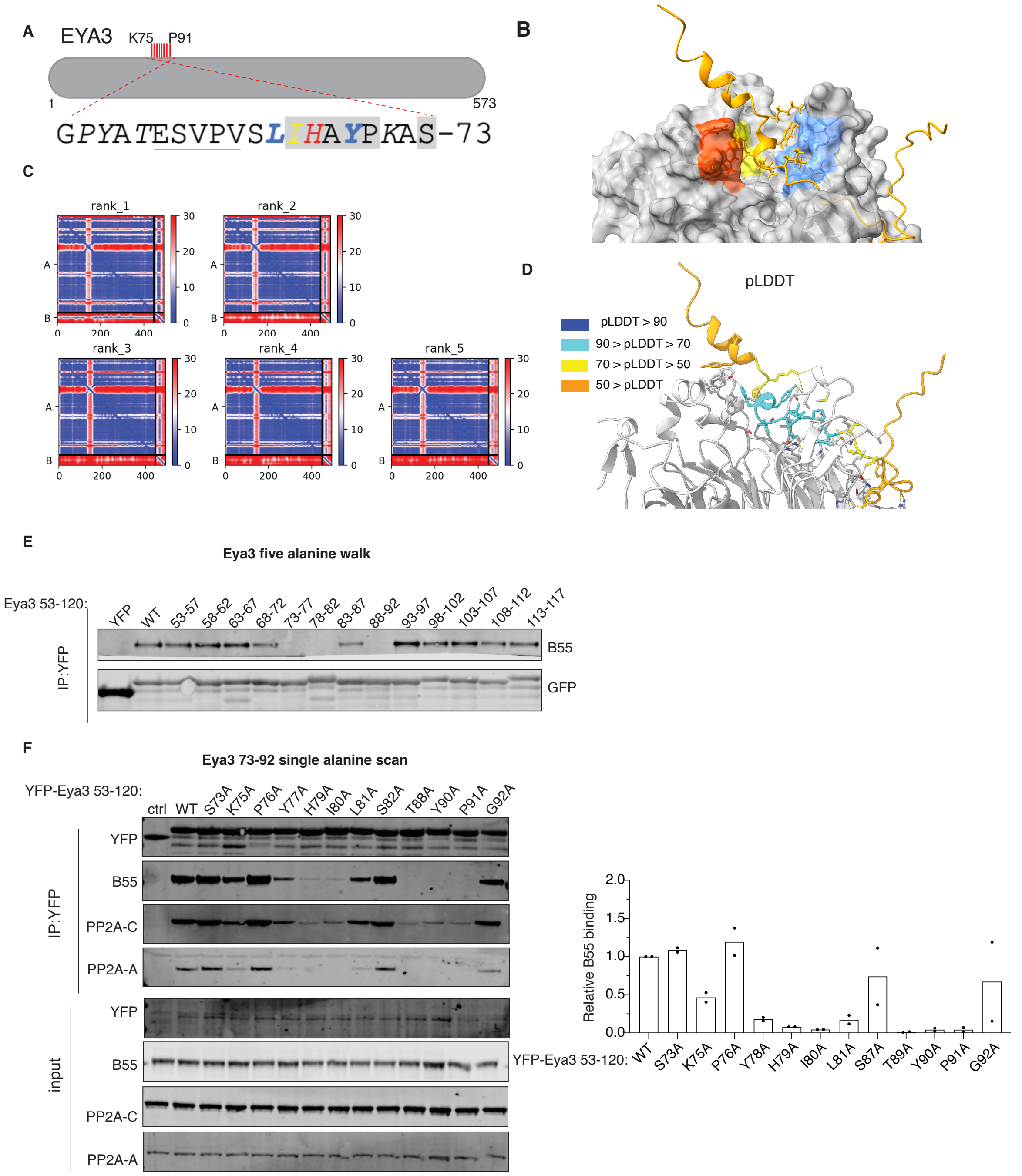

Supplemental Figure S7. EYA3 data.

A) Schematic of EYA3 and sequence binding PP2A-B55α. **B**) AF2 model of EYA binding helix bound to B55. **C**) Predicted aligned error (PAE) score for the 5 models. **D**) pLDDT score mapped onto model. **E-F**) The indicated YFP tagged protein fragments were expressed in HeLa cells and following immunoprecipitation they were probed for binding to PP2A-B55 by western blot. B55 refers to B55α. In F) The quantification of two biological replicates is shown with binding normalized to wild type protein. Bars visualize the average of the two biological replicates.

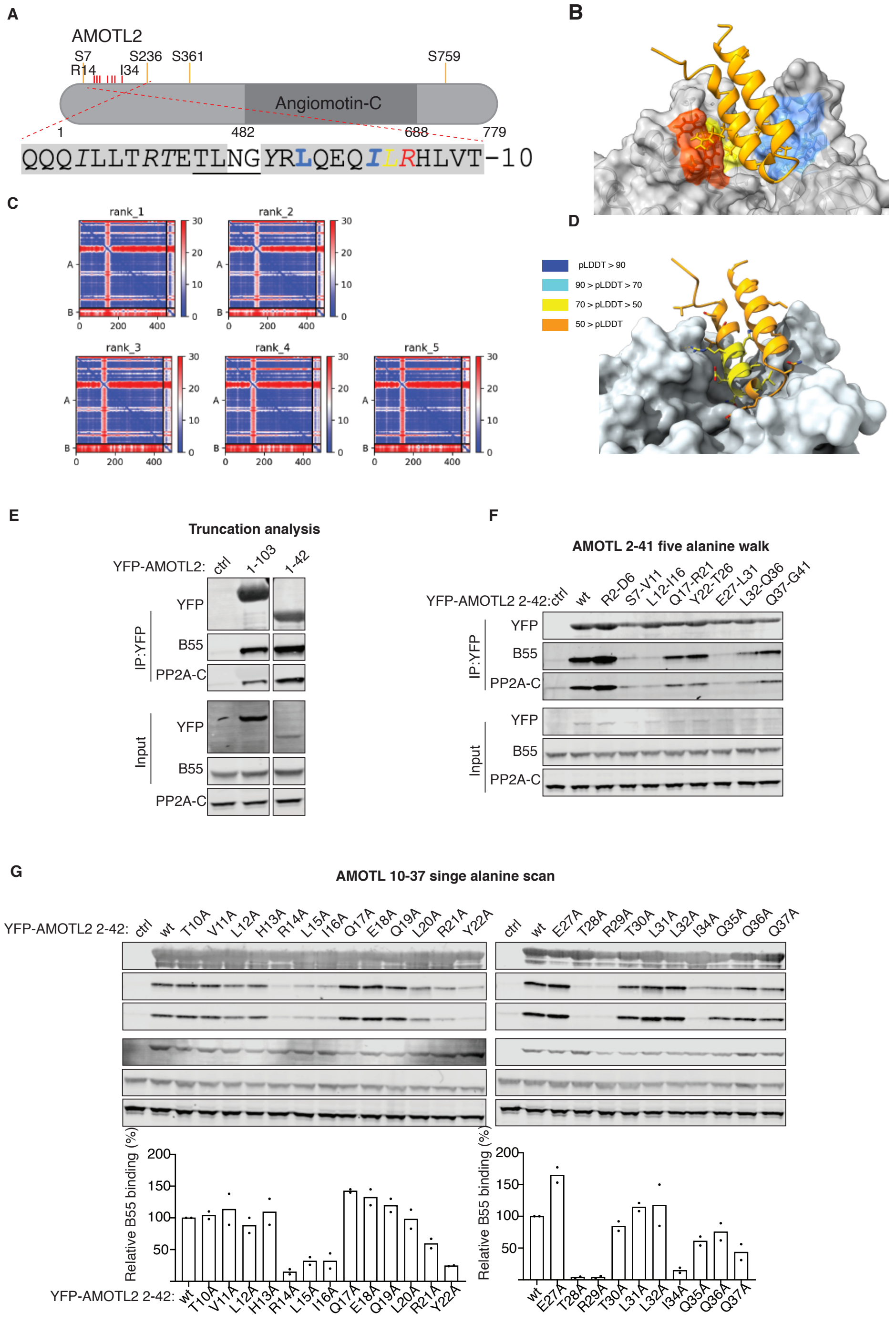

Supplemental Figure S8. AMOTL2 data.

A) Schematic of AMOTL2 and sequence binding PP2A-B55α. Amino acids crucial for binding are indicated by a red line in the schematic. Phosphorylation sites with higher occupancy in AMOTL2 T28A/R29A indicated with yellow lines and amino acid number **B**) AF2 model of AMOTL2 bound to B55. **C**) Predicted aligned error (PAE) score for the 5 models. **D**) pLDDT score mapped onto model. **E-G**) The indicated YFP tagged protein fragments were expressed in HeLa cells and following immunoprecipitation they were probed for binding to PP2A-B55 by western blot. B55 refers to B55α. The quantification of two biological replicates is shown with binding normalized to wild type protein. Bars visualize the average of the two biological replicates.

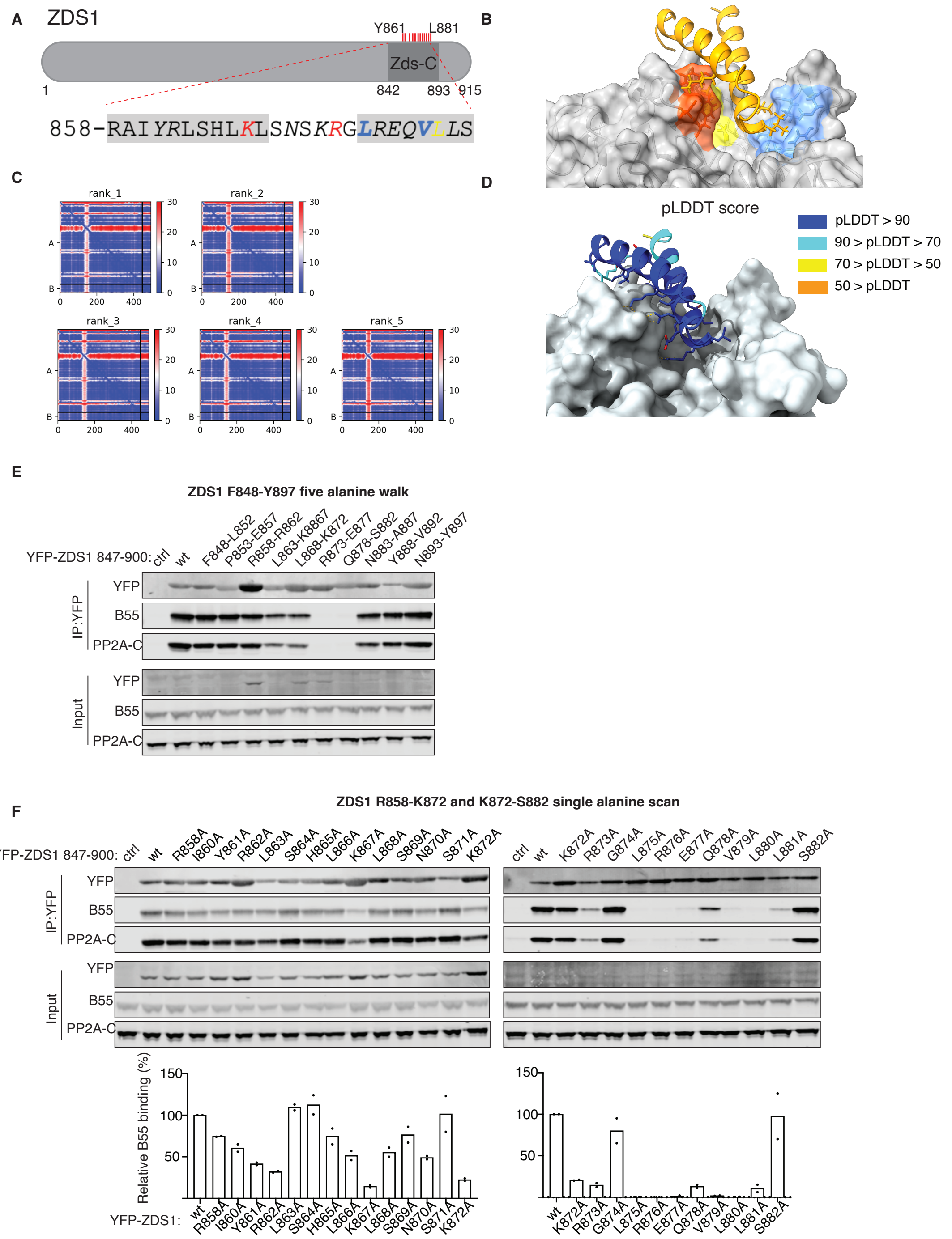

Supplemental Figure S9. Zds1 data.

A) Schematic of Zds1 and sequence binding PP2A-B55α. Amino acids crucial for binding are indicated by a red line in the schematic. **B**) AF2 model of Zds1 binding helixes bound to B55. **C**) Predicted aligned error (PAE) score for the 5 models. **D**) pLDDT score mapped onto model. **E-F**) The indicated YFP tagged protein fragments were expressed in HeLa cells and following immunoprecipitation they were probed for binding to PP2A-B55 by western blot. B55 refers to B55α. In F) The quantification of two biological replicates is shown with binding normalized to wild type protein. Bars visualize the average of the two biological replicates.

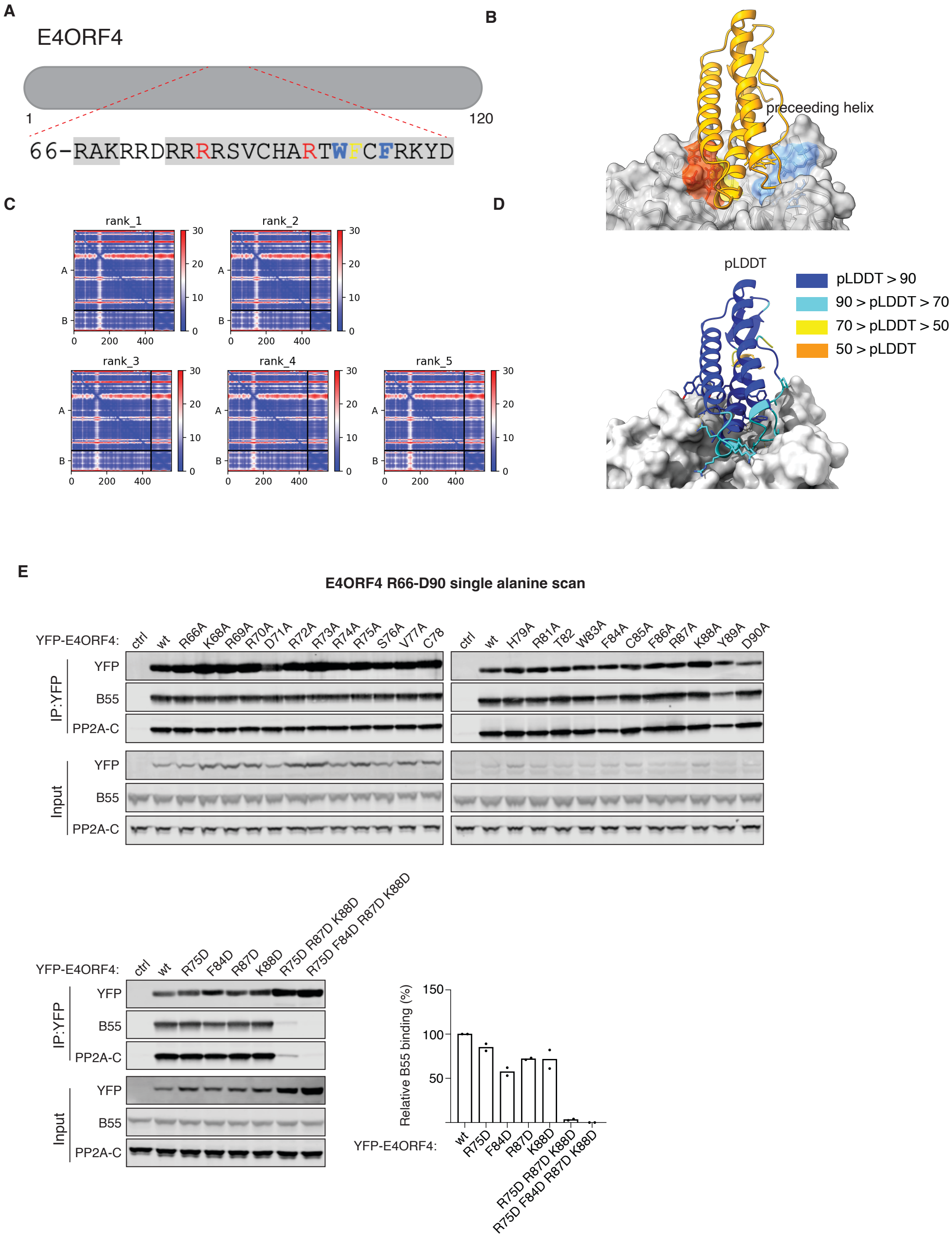

Supplemental Figure S10. E4ORF4 data.

A) Schematic of E4ORF4 and sequence binding PP2A-B55. **B**) AF2 model of E4ORF4 bound to B55. **C**) Predicted aligned error (PAE) score for the 5 models. **D**) pLDDT score mapped onto model. **E)** The indicated YFP tagged protein fragments were expressed in HeLa cells and following immunoprecipitation they were probed for binding to PP2A-B55 by western blot. B55 refers to B55α. **E)** Bottom panel the quantification of two biological replicates is shown with binding normalized to wild type protein. Bars visualize the average of the two biological replicates.

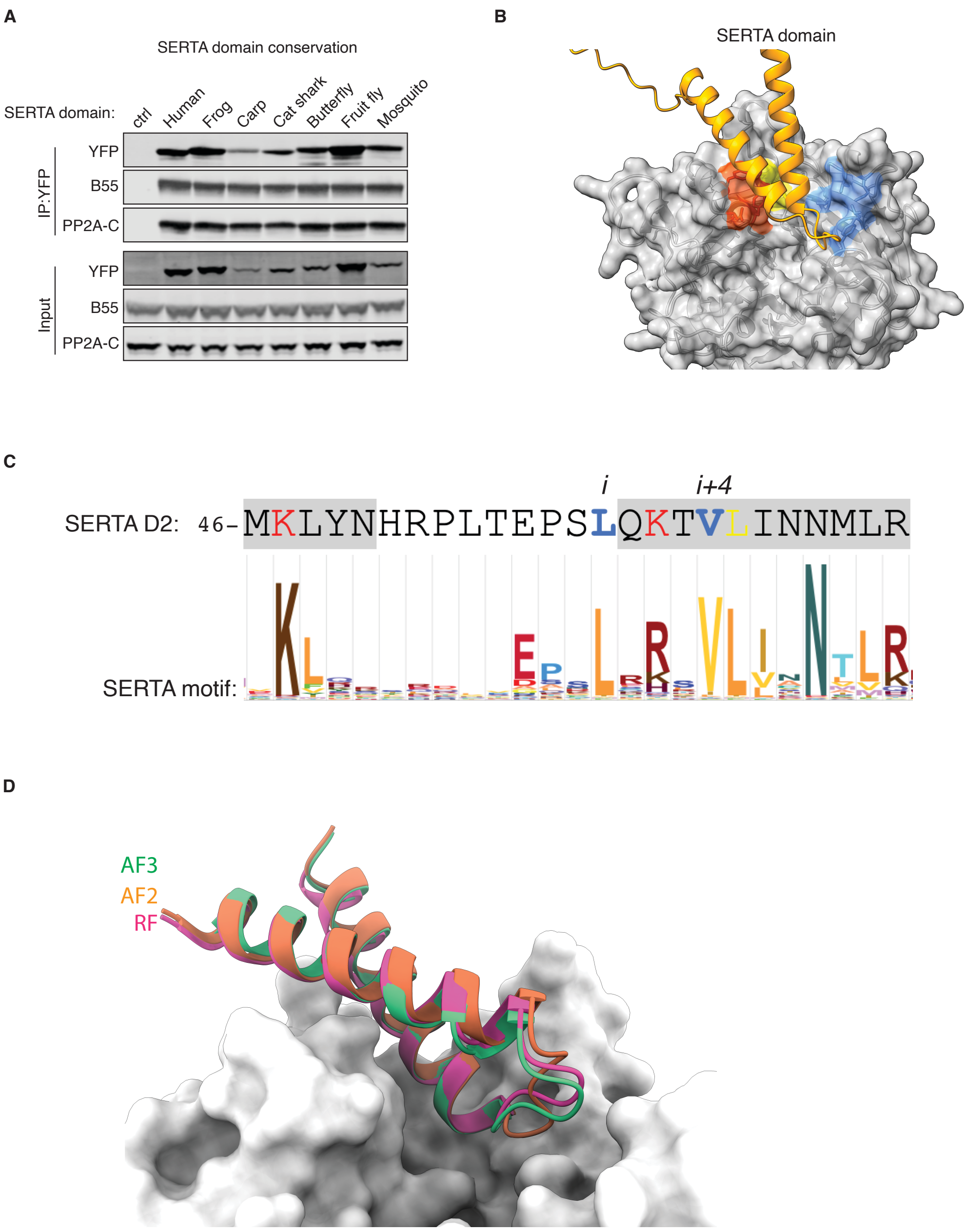

Supplemental Figure S11. Conservation of SERTA domains.
A) YFP tagged SERTA domains from different species were expressed in HeLa cells and binding to PP2A-B55 determined by western-blot. B55 refers to B55α. **B**) AF2 model of the CDCA4 SERTA domain bound to B55. **C**) Signature of all SERTA domains in the Pfam database showing conservation of key residues (data from Pfam entry 06031) **D**) AF2, AF3 and RosettaFold (RF) predictions of CDCA4 bound to B55. Models are overlayed.

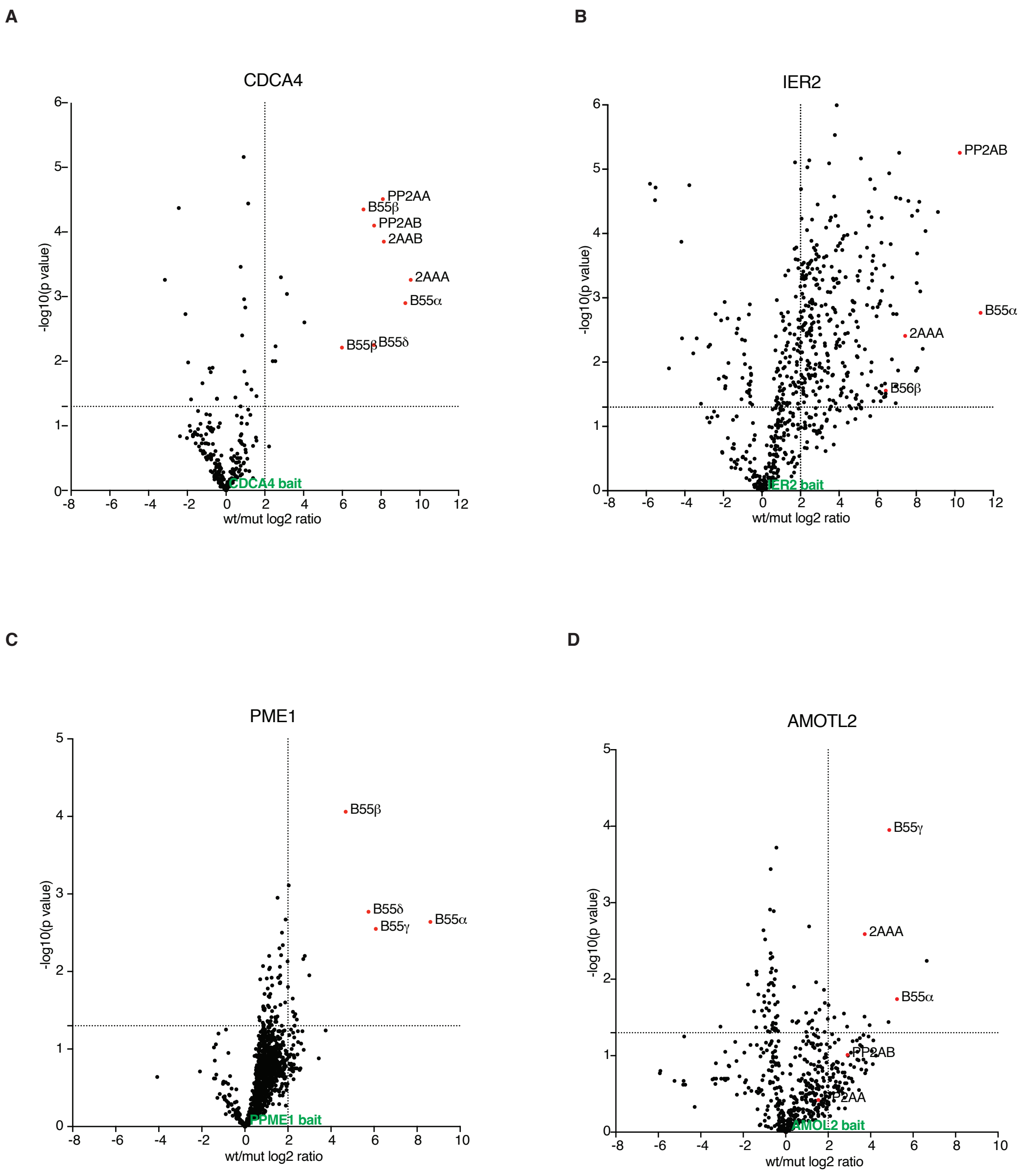

Supplemental Figure S12. Volcano plots of MS data.

A-D) Volcano plots of mass spectrometry analysis of immunopurifications (n=4 technical replicates) of the indicated proteins comparing wild type to a mutant form with the key B55 interacting residues mutated (CDCA4 K44A/V60A/L61A; AMOTL2 T28A/R29A; PME1 L4A; IER2 K17A). PP2AA/B: catalytic subunit, 2AAA: scaffold subunit. See supplemental Table S3 for data.

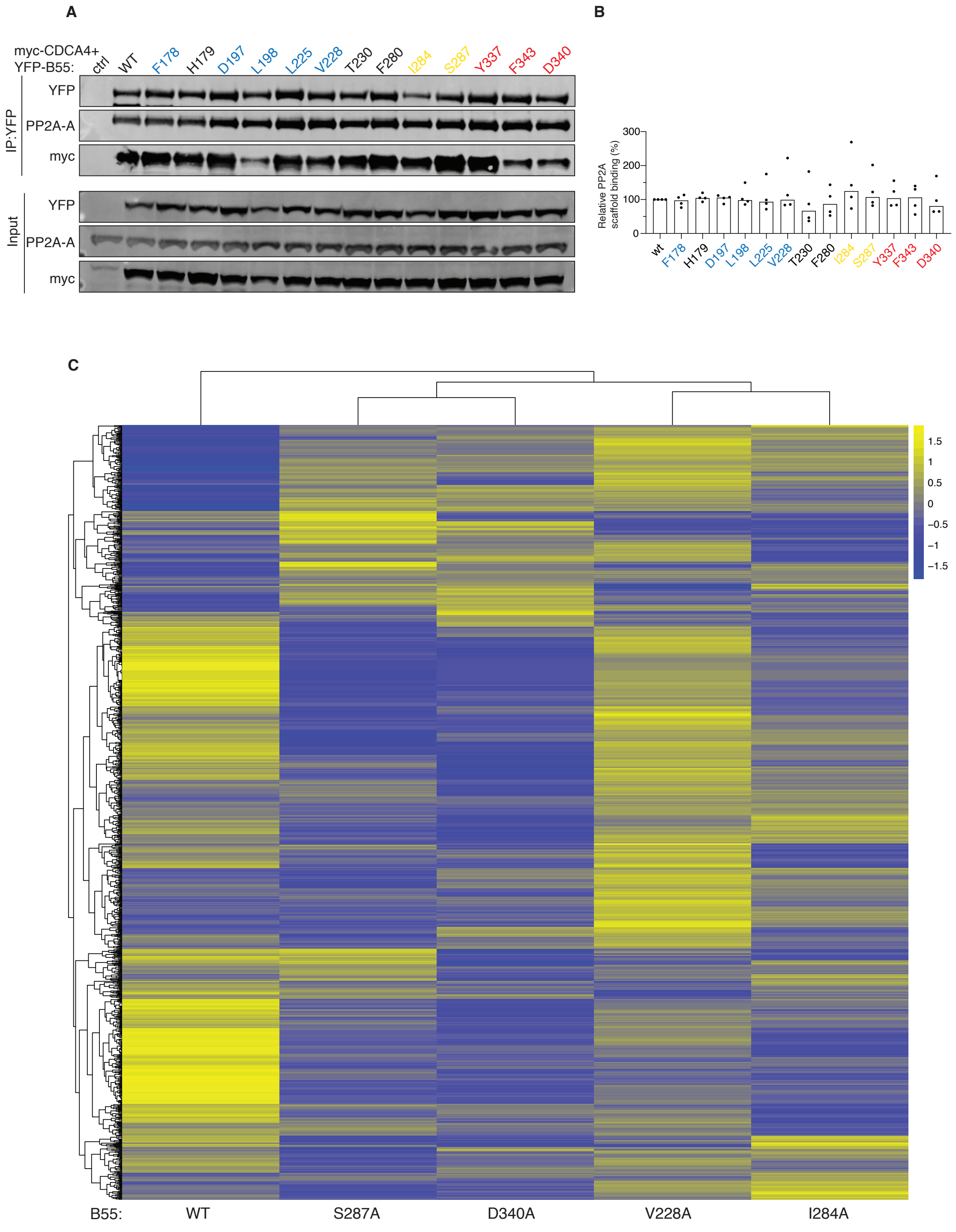

Supplemental Figure S13. **B55 mutant IPs with CDCA4.**

A) Immunoprecipitation of indicated YFP tagged B55α mutants. Binding to myc-CDCA4 determined by western blot **B)** Quantification of A) Relative binding of PP2A scaffold and B55α to CDCA4; median shown, n=4 biological replicates. **C**) HEAT map relating to Figure 2 showing all proteins identified by mass spectrometry for the indicated B55α mutants. Scale bar is log2 and n=4 technical replicates. See Supplemental Table S3 for full data.

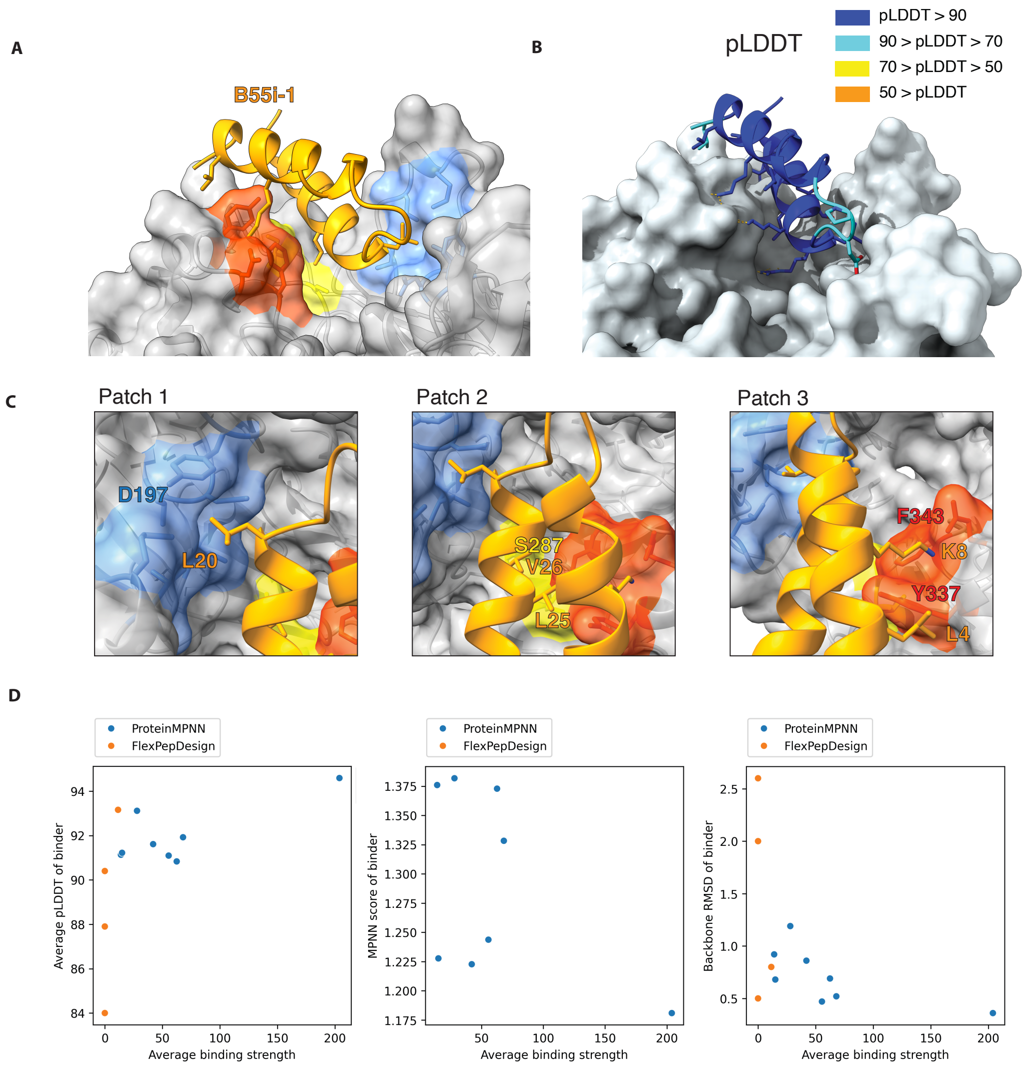

E

Design	Red: loop, Blue: i and i+4	Design strategy
CDCA4 WT	SLQRQSLLDMSLVKLQLCHMLVEPNLCRSVLIANTVRQIQEEM	
B55i-1	------LLELAKKKLKELEEEPDPDLRKKTLVRNMIKKLE---	ProteinMPNN
B55i-2	------EEVERLLKLAEEKLKDESLSLTKKVLLRNLIESI---	ProteinMPNN
B55i-8	------LLELAKKKYEEELKKKEPDLTKLVLLKNVIKKLK---	ProteinMPNN
B55i-12	SLIWQSNMDKALVKLQLCHMLEQPDLTKSVMILAGVLAAQEFM	FlexPepDesign
ZDS1 WT	----NVERAIYRLSHLKLSNSKRGLREQVLLSNFMYAY-------	
B55i-4	----PVKLQLLKKAKEKLADENLSLEKKVLLKNLIKSW-------	ProteinMPNN
B55i-7	----ELIKRLYEKAKEKLKDESRSLEKKVLLRNWIKSL-------	ProteinMPNN
B55i-3	----EEKLRILEKMEEKLKDKSLSLEKKVLLKNAKKSL-------	ProteinMPNN
B55i-5	----EKRKKLKEKALEKLKDPNRDLEEKVLLRNWLKSL-------	ProteinMPNN
B55i-9	RLPINLERALYRLSHLKLSNSKRELYEQVALKLAMLGFLNLVNHT	FlexPepDesign
B55i-10	MQPINKARALYRISHLKLSNSKREQDEQVLLRLHMIAFLNIVNHT	FlexPepDesign
IER2 WT	QRIMTLSVWKMYHSRMQRGGLRLHRSLQLSLVMRSA	
B55i-6	EKILEIAKKKIEELKKKPGGLDLEKYLLYKKLIEKV	ProteinMPNN
B55i-11	QRLMTLSVWKMYHSRMQRGGEDAWLSLMLSLAMRLG	FlexPepDesign

Supplemental Figure S14. B55i and Protein MPNN scores in relation to binding. **A-B**) AF model of B55i-1 bound to B55 and pLDDT score. **C**) Zoom in on interactions of B55i-1 with the different patches. **D**) Plots of design scores and binding strength based on immunopurifications from HeLa cells of YFP tagged de novo B55 binders. **E)** Amino acid sequence of MPNN and FlexPep generated B55 binding designs.

F

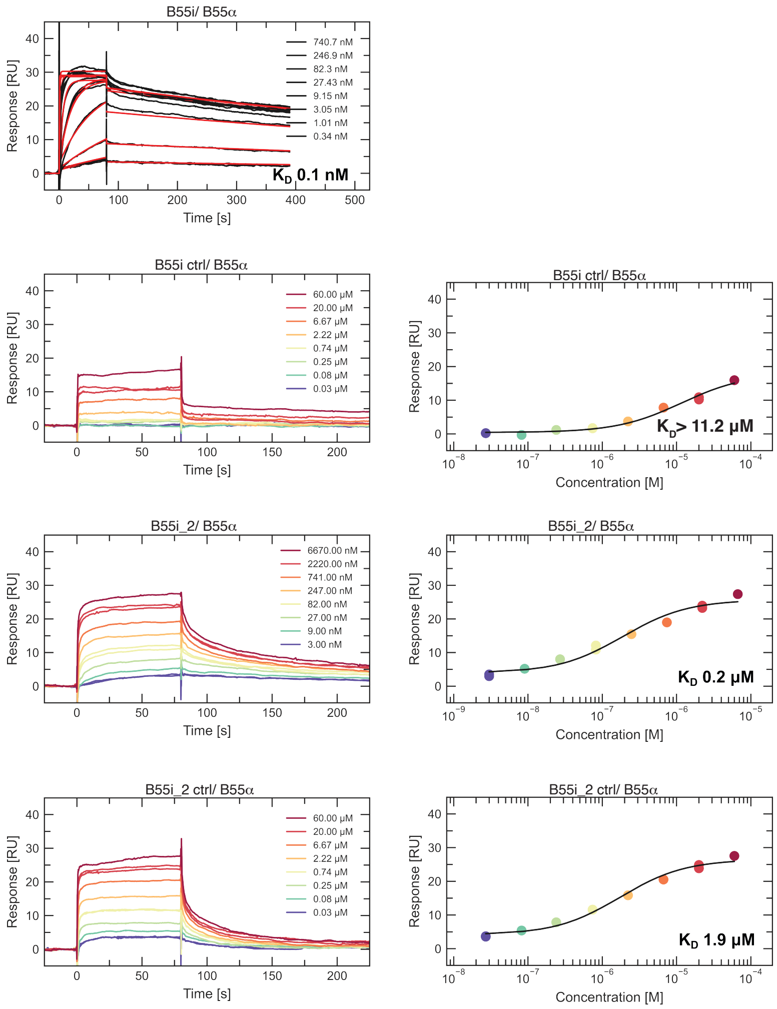

G

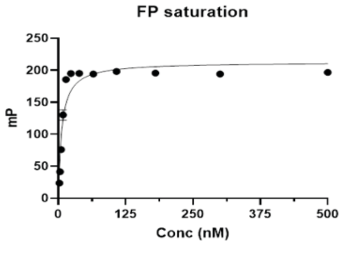

H

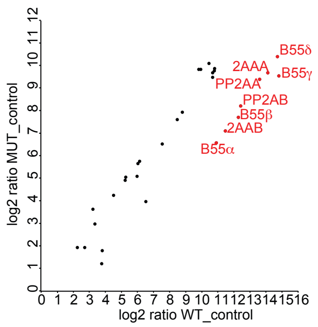

Supplemental Figure S14-continued. SPR and MS data for B55i.

F) SPR data for B55i-1 and B55i-2 peptides binding to B55. **G**) FP measurements for B55i (n=3). **H**) Mass spectrometry analysis of B55i and B55i CTRL based on n=4 technical replicates.

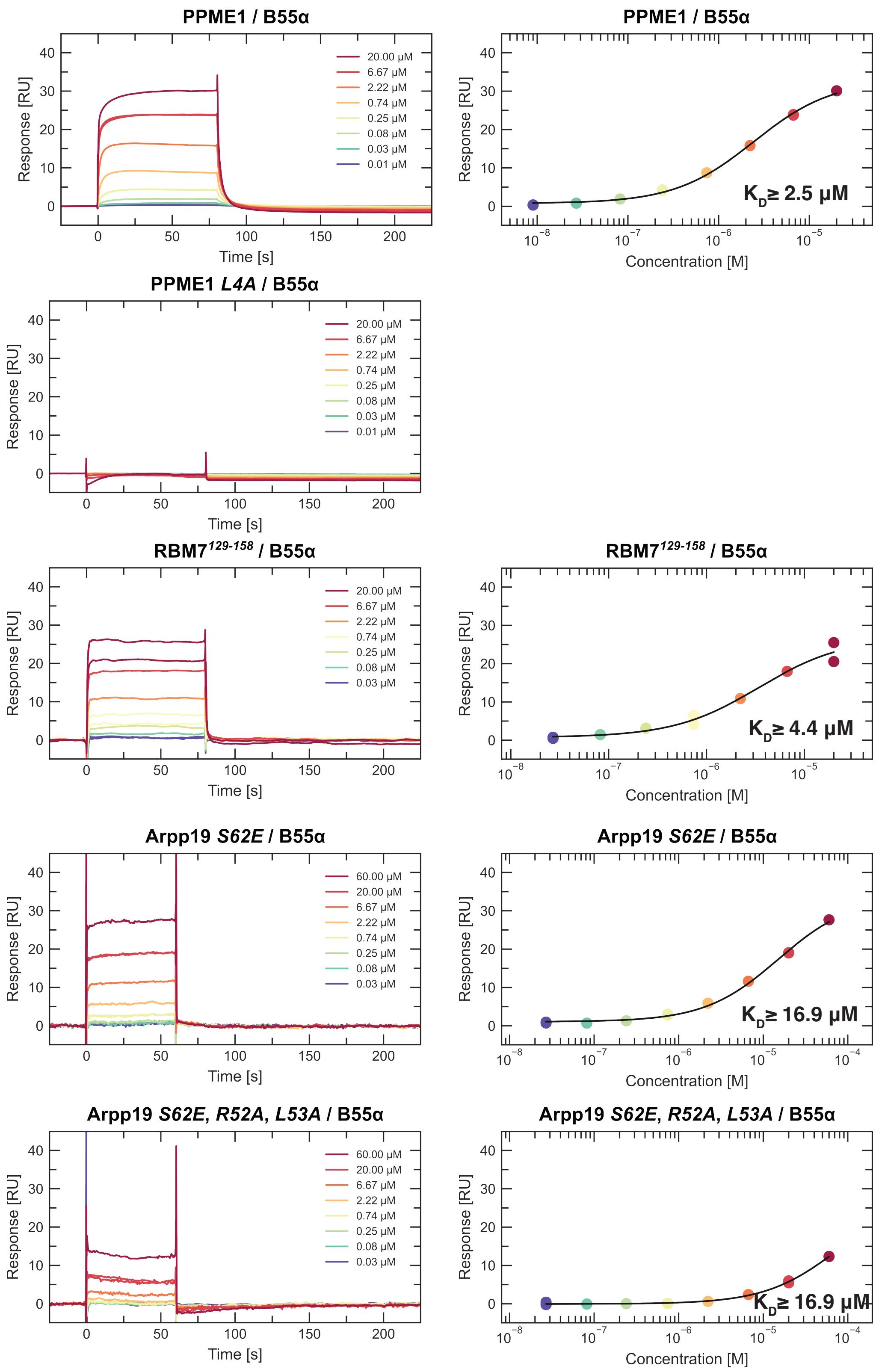

Supplemental Figure S15. SPR data for PME1, Arpp19 and RBM7.
SPR data for the indicated proteins binding to B55.

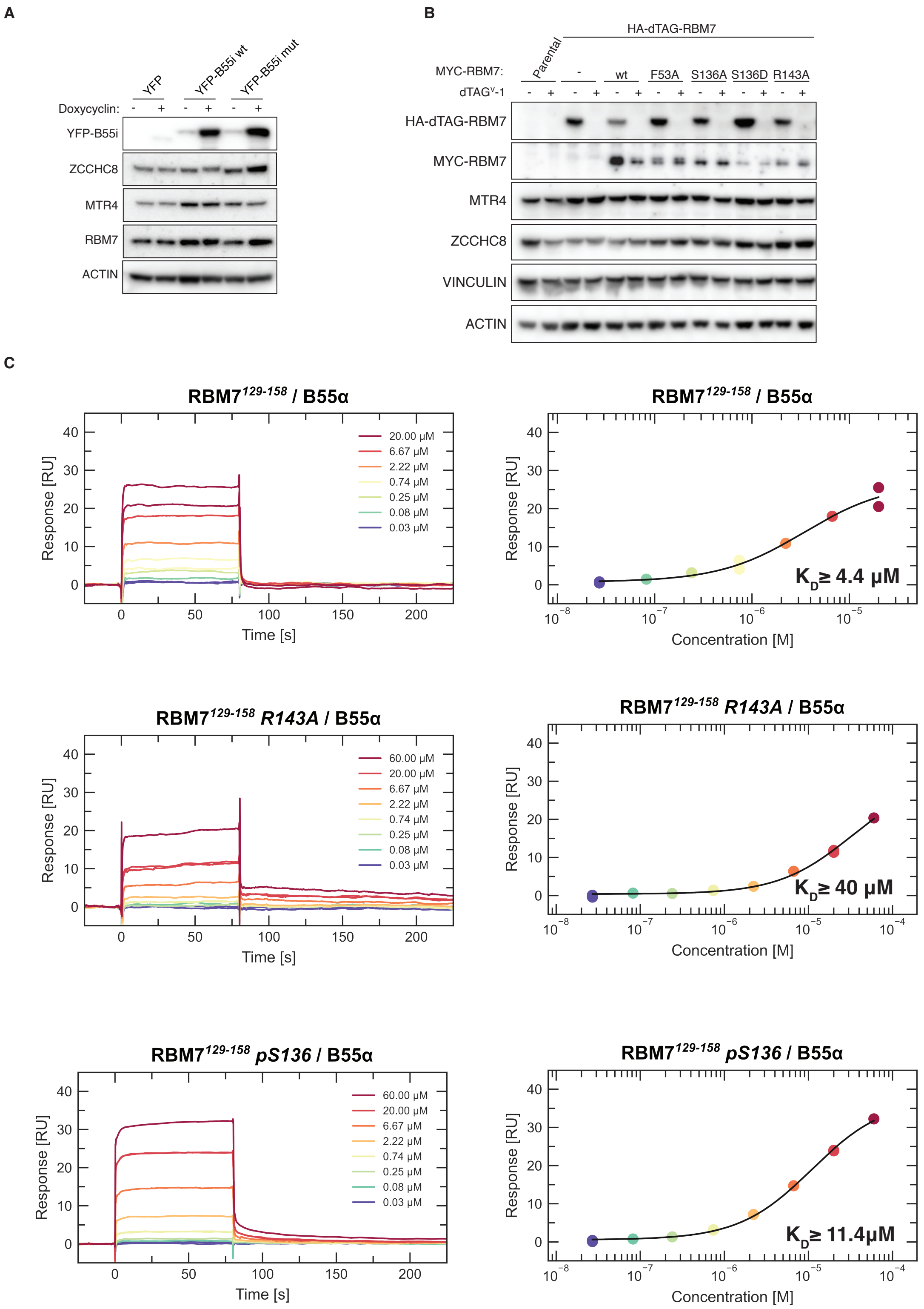

Supplemental Figure S16. Western blots relating to RBM7 biology and RBM7 SPR data.

A) Western blots of B55i-1 expression from inducible HeLa cells. **B**) Removal of HA-dTAG-RBM7 with dTAG-1 and complementation with myc-RBM7 variants. **C**) SPR data for the indicated RBM7 peptides binding to B55.

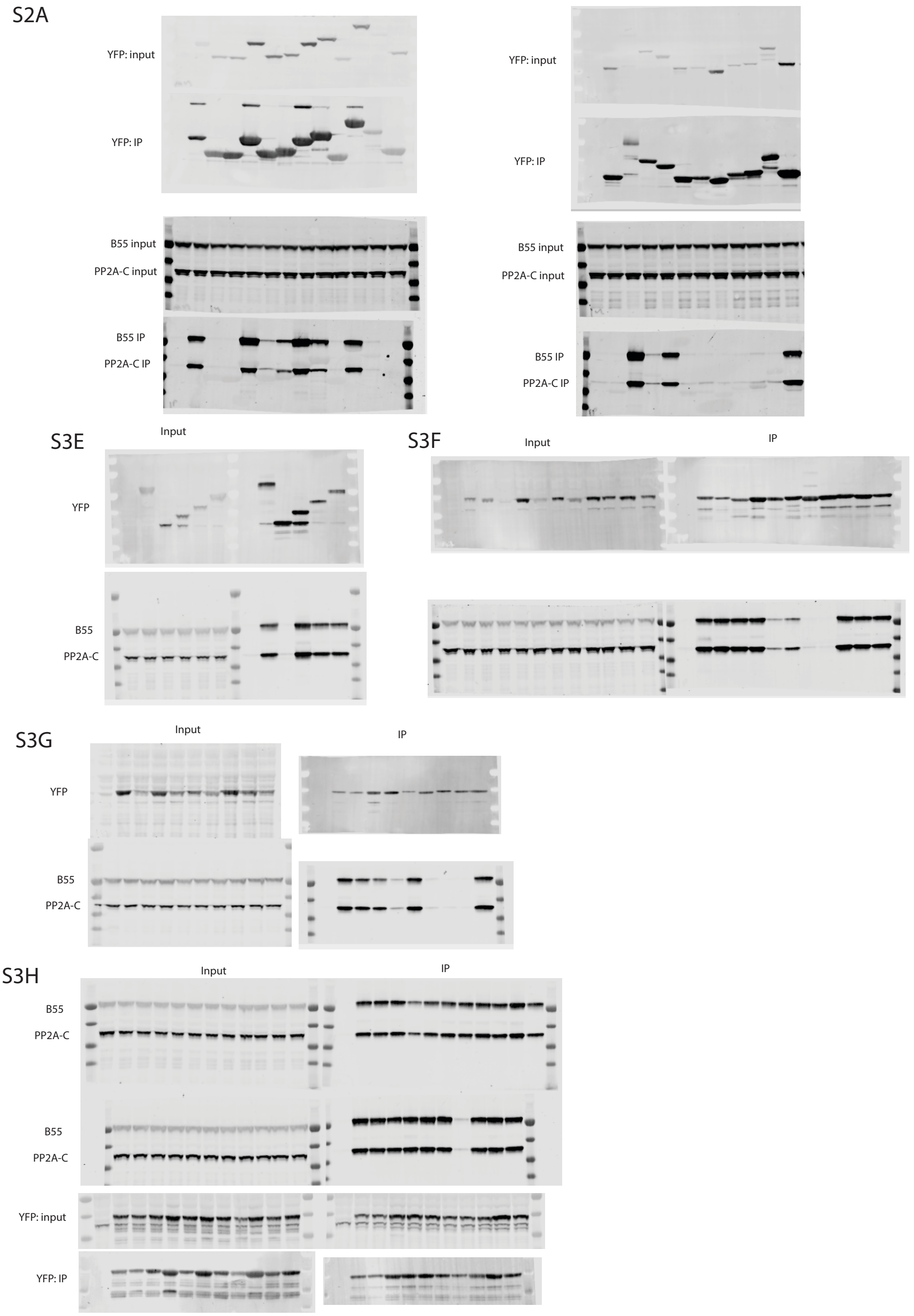

Supplemental Figure S17. Uncropped western blots of selected panels.

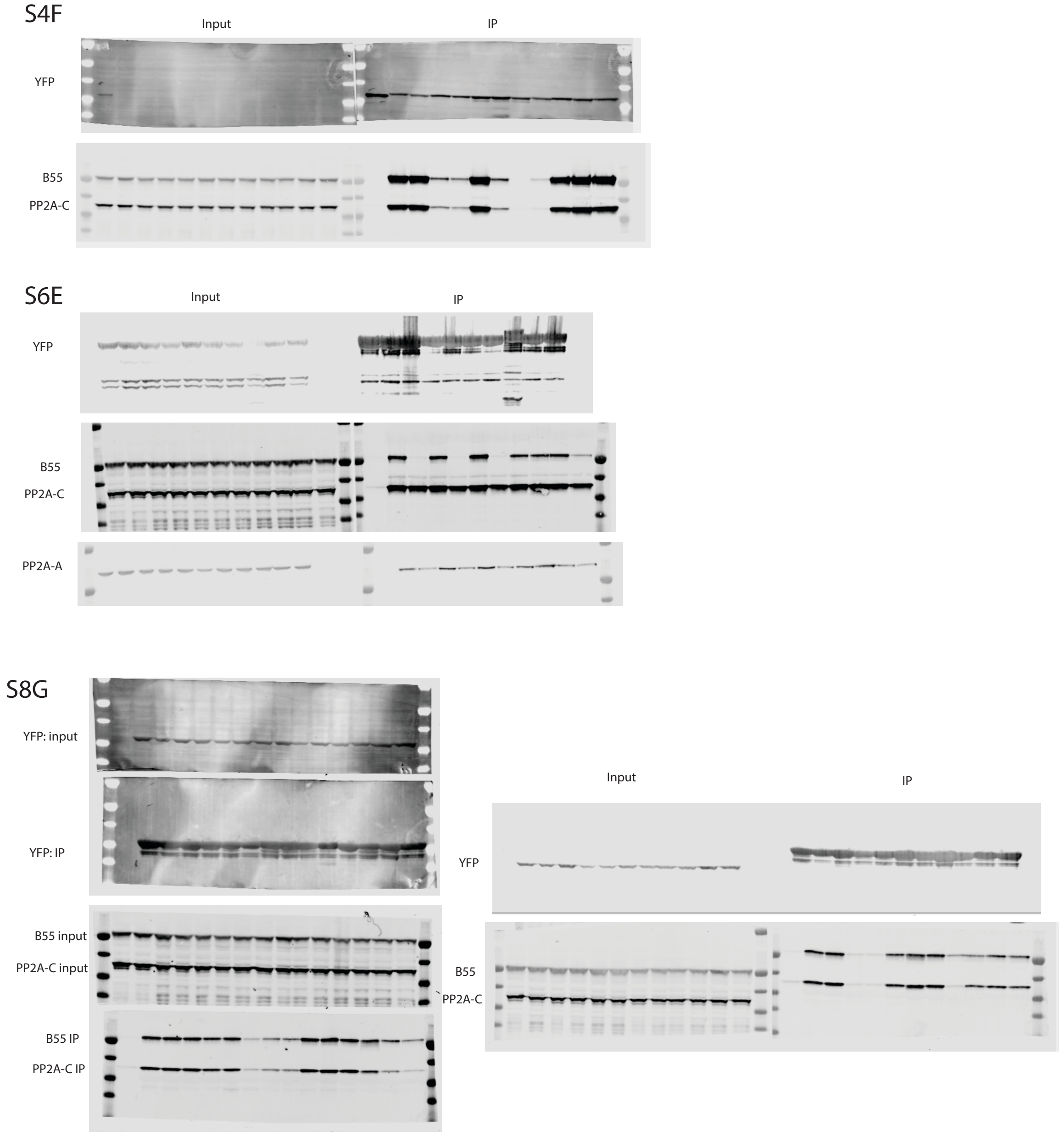

Supplemental Figure S17 continued. Uncropped western blots of selected panels.

S9F

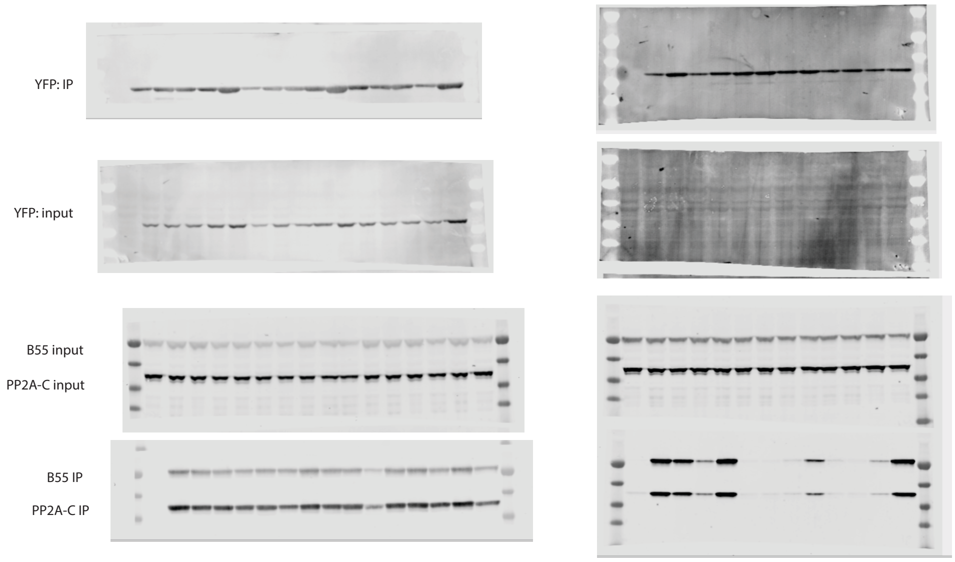

S10E

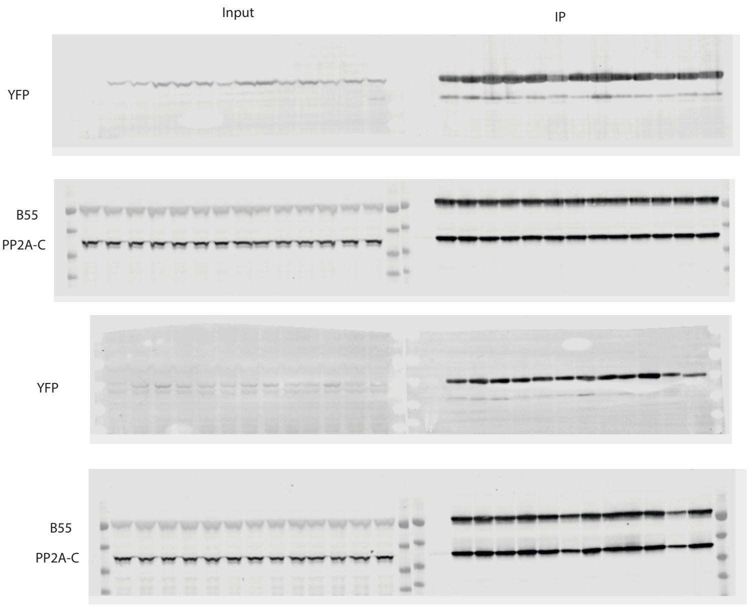

Supplemental Figure S17 continued. Uncropped western blots of selected panels.

af2_scores

pdb int_plddt iPAE
b55_A1X283-SPD2B_HUMAN_unrelaxed_rank_001_alphafold2_multimer_v3_model_1_seed_000.pdb 73.73 9.26
b55_A2A3K4-PTPC1_HUMAN_unrelaxed_rank_001_alphafold2_multimer_v3_model_1_seed_000.pdb 0.0 24.55
b55_A3KMH1-VWA8_HUMAN_unrelaxed_rank_001_alphafold2_multimer_v3_model_5_seed_000.pdb 65.31 26.28
b55_A3KN83-SBNO1_HUMAN_unrelaxed_rank_001_alphafold2_multimer_v3_model_4_seed_000.pdb 0.0 22.28
b55_A7KAX9-RHG32_HUMAN_unrelaxed_rank_001_alphafold2_multimer_v3_model_3_seed_000.pdb 58.69 15.41
b55_A8MPP1-D11L8_HUMAN_unrelaxed_rank_001_alphafold2_multimer_v3_model_4_seed_000.pdb 72.52 27.19
b55_O00165-HAX1_HUMAN_unrelaxed_rank_001_alphafold2_multimer_v3_model_1_seed_000.pdb 0 30
b55_O00311-CDC7_HUMAN_unrelaxed_rank_004_alphafold2_multimer_v3_model_2_seed_000.pdb 62.44 22.7
b55_O00505-IMA4_HUMAN_unrelaxed_rank_003_alphafold2_multimer_v3_model_2_seed_000.pdb 0.0 25.27
b55_O14530-TXND9_HUMAN_unrelaxed_rank_002_alphafold2_multimer_v3_model_3_seed_000.pdb 73.14 26.31
b55_O14734-ACOT8_HUMAN_unrelaxed_rank_003_alphafold2_multimer_v3_model_1_seed_000.pdb 0 18.86
b55_O14737-PDCD5_HUMAN_unrelaxed_rank_001_alphafold2_multimer_v3_model_4_seed_000.pdb 84.41 4.27
b55_O14795-UN13B_HUMAN_unrelaxed_rank_001_alphafold2_multimer_v3_model_2_seed_000.pdb 65.47 10.72
b55_O14907-TX1B3_HUMAN_unrelaxed_rank_001_alphafold2_multimer_v3_model_3_seed_000.pdb 71.1 22.62
b55_O15061-SYNEM_HUMAN_unrelaxed_rank_001_alphafold2_multimer_v3_model_1_seed_000.pdb 0 30
b55_O43166-SI1L1_HUMAN_unrelaxed_rank_001_alphafold2_multimer_v3_model_1_seed_000.pdb 69.19 21.58
b55_O43593-HAIR_HUMAN_unrelaxed_rank_001_alphafold2_multimer_v3_model_4_seed_000.pdb 65.95 8.05
b55_O43768-ENSA_HUMAN_unrelaxed_rank_002_alphafold2_multimer_v3_model_5_seed_000.pdb 77.41 5.06
b55_O43819-SCO2_HUMAN_unrelaxed_rank_001_alphafold2_multimer_v3_model_2_seed_000.pdb 0.0 14.35
b55_O60343-TBCD4_HUMAN_unrelaxed_rank_001_alphafold2_multimer_v3_model_1_seed_000.pdb 64.14 14.22
b55_O60548-FOXD2_HUMAN_unrelaxed_rank_002_alphafold2_multimer_v3_model_5_seed_000.pdb 79.68 6.23
b55_O60610-DIAP1_HUMAN_unrelaxed_rank_001_alphafold2_multimer_v3_model_2_seed_000.pdb 0 30
b55_O60884-DNJA2_HUMAN_unrelaxed_rank_001_alphafold2_multimer_v3_model_1_seed_000.pdb 0 30
b55_O60925-PFD1_HUMAN_unrelaxed_rank_003_alphafold2_multimer_v3_model_4_seed_000.pdb 0 12.97
b55_O75190-DNJB6_HUMAN_unrelaxed_rank_005_alphafold2_multimer_v3_model_2_seed_000.pdb 64.31 23.16
b55_O75376-NCOR1_HUMAN_unrelaxed_alphafold2_multimer_v3_model_2_seed_000.pdb 0 12.58
b55_O75665-OFD1_HUMAN_unrelaxed_rank_001_alphafold2_multimer_v3_model_1_seed_000.pdb 0 30
b55_O75792-RNH2A_HUMAN_unrelaxed_rank_001_alphafold2_multimer_v3_model_5_seed_000.pdb 0 30
b55_O94763-RMP_HUMAN_unrelaxed_rank_004_alphafold2_multimer_v3_model_1_seed_000.pdb 0.0 26.75
b55_O95071-UBR5_HUMAN_unrelaxed_alphafold2_multimer_v3_model_1_seed_000.pdb 0 30
b55_O95263-PDE8B_HUMAN_unrelaxed_rank_004_alphafold2_multimer_v3_model_2_seed_000.pdb 77.47 26.86
b55_O95816-BAG2_HUMAN_unrelaxed_rank_002_alphafold2_multimer_v3_model_1_seed_000.pdb 63.8 30
b55_O95817-BAG3_HUMAN_unrelaxed_rank_001_alphafold2_multimer_v3_model_4_seed_000.pdb 61.61 25.19
b55_P01215-GLHA_HUMAN_unrelaxed_rank_004_alphafold2_multimer_v3_model_3_seed_000.pdb 0 24.62
b55_P04792-HSPB1_HUMAN_unrelaxed_rank_001_alphafold2_multimer_v3_model_2_seed_000.pdb 0.0 7.35
b55_P07900-HS90A_HUMAN_unrelaxed_rank_001_alphafold2_multimer_v3_model_5_seed_000.pdb 62.79 22.92
b55_P08238-HS90B_HUMAN_unrelaxed_rank_001_alphafold2_multimer_v3_model_5_seed_000.pdb 66.88 22.44
b55_P0CG12-DERPC_HUMAN_unrelaxed_rank_001_alphafold2_multimer_v3_model_5_seed_000.pdb 82.4 3.89

Supplemental Table S1. **AF2 scores for models with pLDDT and iPAE indicated after pdb file.**

b55_P11498-PYC_HUMAN_unrelaxed_rank_003_alphafold2_multimer_v3_model_4_seed_000.pdb 72.93 26.23
b55_P12036-NFH_HUMAN_unrelaxed_rank_001_alphafold2_multimer_v3_model_2_seed_000.pdb 0 30
b55_P12270-TPR_HUMAN_unrelaxed_rank_001_alphafold2_multimer_v3_model_1_seed_000.pdb 0 30
b55_P13051-UNG_HUMAN_unrelaxed_rank_002_alphafold2_multimer_v3_model_5_seed_000.pdb 79.4 25.72
b55_P17987-TCPA_HUMAN_unrelaxed_rank_001_alphafold2_multimer_v3_model_4_seed_000.pdb 0 30
b55_P19387-RPB3_HUMAN_unrelaxed_rank_001_alphafold2_multimer_v3_model_5_seed_000.pdb 0 30
b55_P23458-JAK1_HUMAN_unrelaxed_rank_003_alphafold2_multimer_v3_model_4_seed_000.pdb 67.14 27.44
b55_P27540-ARNT_HUMAN_unrelaxed_rank_001_alphafold2_multimer_v3_model_2_seed_000.pdb 57.84 15.93
b55_P27708-PYR1_HUMAN_unrelaxed_rank_003_alphafold2_multimer_v3_model_4_seed_000.pdb 75.68 27.66
b55_P29218-IMPA1_HUMAN_unrelaxed_rank_001_alphafold2_multimer_v3_model_3_seed_000.pdb 72.58 26.89
b55_P30876-RPB2_HUMAN_unrelaxed_rank_001_alphafold2_multimer_v3_model_2_seed_000.pdb 0 30
b55_P31689-DNJA1_HUMAN_unrelaxed_rank_001_alphafold2_multimer_v3_model_3_seed_000.pdb 66.39 26.02
b55_P31948-STIP1_HUMAN_unrelaxed_rank_002_alphafold2_multimer_v3_model_1_seed_000.pdb 63.17 22.95
b55_P32926-DSG3_HUMAN_unrelaxed_rank_001_alphafold2_multimer_v3_model_4_seed_000.pdb 84.95 5.93
b55_P36873-PP1G_HUMAN_unrelaxed_rank_002_alphafold2_multimer_v3_model_1_seed_000.pdb 78.52 19.84
b55_P40227-TCPZ_HUMAN_unrelaxed_rank_002_alphafold2_multimer_v3_model_3_seed_000.pdb 0 26.66
b55_P42345-MTOR_HUMAN_unrelaxed_alphafold2_multimer_v3_model_1_seed_000.pdb 83.96 27.75
b55_P48552-NRIP1_HUMAN_unrelaxed_rank_001_alphafold2_multimer_v3_model_4_seed_000.pdb 53.92 12.51
b55_P48643-TCPE_HUMAN_unrelaxed_rank_001_alphafold2_multimer_v3_model_1_seed_000.pdb 0 16.91
b55_P48681-NEST_HUMAN_unrelaxed_rank_001_alphafold2_multimer_v3_model_4_seed_000.pdb 0 30
b55_P49368-TCPG_HUMAN_unrelaxed_rank_001_alphafold2_multimer_v3_model_2_seed_000.pdb 0 30
b55_P50990-TCPQ_HUMAN_unrelaxed_rank_001_alphafold2_multimer_v3_model_2_seed_000.pdb 0.0 25.45
b55_P50991-TCPD_HUMAN_unrelaxed_rank_004_alphafold2_multimer_v3_model_4_seed_000.pdb 0 26.36
b55_P53041-PPP5_HUMAN_unrelaxed_rank_001_alphafold2_multimer_v3_model_1_seed_000.pdb 68.24 24.08
b55_P53634-CATC_HUMAN_unrelaxed_rank_002_alphafold2_multimer_v3_model_1_seed_000.pdb 66.76 26.22
b55_P53814-SMTN_HUMAN_unrelaxed_rank_002_alphafold2_multimer_v3_model_5_seed_000.pdb 70.95 20.64
b55_P55789-ALR_HUMAN_unrelaxed_rank_001_alphafold2_multimer_v3_model_2_seed_000.pdb 0 11.52
b55_P56211-ARP19_HUMAN_unrelaxed_rank_005_alphafold2_multimer_v3_model_4_seed_000.pdb 77.56 4.71
b55_P56524-HDAC4_HUMAN_unrelaxed_rank_001_alphafold2_multimer_v3_model_1_seed_000.pdb 54.55 12.74
b55_P60510-PP4C_HUMAN_unrelaxed_rank_001_alphafold2_multimer_v3_model_3_seed_000.pdb 78.39 8.01
b55_P61758-PFD3_HUMAN_unrelaxed_rank_004_alphafold2_multimer_v3_model_4_seed_000.pdb 65.94 30
b55_P62136-PP1A_HUMAN_unrelaxed_rank_002_alphafold2_multimer_v3_model_1_seed_000.pdb 79.75 22.11
b55_P62140-PP1B_HUMAN_unrelaxed_rank_001_alphafold2_multimer_v3_model_5_seed_000.pdb 0.0 13.51
b55_P63151-2ABA_HUMAN_unrelaxed_rank_002_alphafold2_multimer_v3_model_3_seed_000.pdb 72.77 23.09
b55_P78371-TCPB_HUMAN_unrelaxed_rank_003_alphafold2_multimer_v3_model_4_seed_000.pdb 65.46 26.69
b55_P86791-CCZ1_HUMAN_unrelaxed_rank_003_alphafold2_multimer_v3_model_4_seed_000.pdb 0.0 16.38
b55_Q00005-2ABB_HUMAN_unrelaxed_rank_002_alphafold2_multimer_v3_model_1_seed_000.pdb 75.07 21.14
b55_Q00587-BORG5_HUMAN_unrelaxed_rank_001_alphafold2_multimer_v3_model_4_seed_000.pdb 70.91 7.94
b55_Q05209-PTN12_HUMAN_unrelaxed_rank_002_alphafold2_multimer_v3_model_3_seed_000.pdb 76.67 20.7
b55_Q08209-PP2BA_HUMAN_unrelaxed_rank_003_alphafold2_multimer_v3_model_1_seed_000.pdb 0.0 26.68

Supplemental Table S1 continued. **AF2 scores for models with pLDDT and iPAE indicated after pdb file.**

b55_Q08AF3-SLFN5_HUMAN_unrelaxed_rank_002_alphafold2_multimer_v3_model_1_seed_000.pdb 69.07 27.17
b55_Q0VG06-FP100_HUMAN_unrelaxed_rank_002_alphafold2_multimer_v3_model_2_seed_000.pdb 68.07 26.7
b55_Q12815-TROAP_HUMAN_unrelaxed_rank_001_alphafold2_multimer_v3_model_2_seed_000.pdb 73.87 5.38
b55_Q12851-M4K2_HUMAN_unrelaxed_rank_002_alphafold2_multimer_v3_model_5_seed_000.pdb 71.28 25.25
b55_Q12948-FOXC1_HUMAN_unrelaxed_rank_001_alphafold2_multimer_v3_model_3_seed_000.pdb 84.19 4.39
b55_Q13015-AF1Q_HUMAN_unrelaxed_rank_001_alphafold2_multimer_v3_model_3_seed_000.pdb 0 30
b55_Q13049-TRI32_HUMAN_unrelaxed_rank_004_alphafold2_multimer_v3_model_4_seed_000.pdb 67.48 24.38
b55_Q13322-GRB10_HUMAN_unrelaxed_rank_002_alphafold2_multimer_v3_model_1_seed_000.pdb 68.71 26.3
b55_Q13371-PHLP_HUMAN_unrelaxed_rank_001_alphafold2_multimer_v3_model_3_seed_000.pdb 75.4 7.03
b55_Q13451-FKBP5_HUMAN_unrelaxed_rank_002_alphafold2_multimer_v3_model_2_seed_000.pdb 74.06 19.1
b55_Q13523-PRP4B_HUMAN_unrelaxed_rank_001_alphafold2_multimer_v3_model_1_seed_000.pdb 60.56 12.73
b55_Q13671-RIN1_HUMAN_unrelaxed_rank_001_alphafold2_multimer_v3_model_4_seed_000.pdb 72.99 5.86
b55_Q14140-SRTD2_HUMAN_unrelaxed_rank_003_alphafold2_multimer_v3_model_4_seed_000.pdb 92.53 2.16
b55_Q14678-KANK1_HUMAN_unrelaxed_rank_001_alphafold2_multimer_v3_model_3_seed_000.pdb 0 9.57
b55_Q15031-SYLM_HUMAN_unrelaxed_rank_002_alphafold2_multimer_v3_model_3_seed_000.pdb 75.96 26.16
b55_Q15438-CYH1_HUMAN_unrelaxed_rank_001_alphafold2_multimer_v3_model_2_seed_000.pdb 69.31 11.83
b55_Q15468-STIL_HUMAN_unrelaxed_rank_002_alphafold2_multimer_v3_model_4_seed_000.pdb 0.0 13.74
b55_Q15773-MLF2_HUMAN_unrelaxed_rank_001_alphafold2_multimer_v3_model_2_seed_000.pdb 68.78 9.06
b55_Q16342-PDCD2_HUMAN_unrelaxed_rank_004_alphafold2_multimer_v3_model_5_seed_000.pdb 82.2 24.47
b55_Q16563-SYPL1_HUMAN_unrelaxed_rank_001_alphafold2_multimer_v3_model_2_seed_000.pdb 59.95 12.11
b55_Q16584-M3K11_HUMAN_unrelaxed_rank_001_alphafold2_multimer_v3_model_5_seed_000.pdb 66.74 24.78
b55_Q1MSJ5-CSPP1_HUMAN_unrelaxed_rank_001_alphafold2_multimer_v3_model_2_seed_000.pdb 77.31 5.68
b55_Q2KHR3-QSER1_HUMAN_unrelaxed_rank_002_alphafold2_multimer_v3_model_1_seed_000.pdb 59.27 23.66
b55_Q2M3G4-SHRM1_HUMAN_unrelaxed_rank_003_alphafold2_multimer_v3_model_3_seed_000.pdb 73.59 15.77
b55_Q32MH5-ATOSA_HUMAN_unrelaxed_rank_002_alphafold2_multimer_v3_model_5_seed_000.pdb 89.82 3.27
b55_Q3YEC7-RABL6_HUMAN_unrelaxed_rank_001_alphafold2_multimer_v3_model_4_seed_000.pdb 0.0 17.72
b55_Q53ET0-CRTC2_HUMAN_unrelaxed_rank_001_alphafold2_multimer_v3_model_5_seed_000.pdb 0 30
b55_Q58FF8-H90B2_HUMAN_unrelaxed_rank_002_alphafold2_multimer_v3_model_2_seed_000.pdb 0.0 22.53
b55_Q58WW2-DCAF6_HUMAN_unrelaxed_rank_001_alphafold2_multimer_v3_model_5_seed_000.pdb 0.0 26.14
b55_Q5M775-CYTSB_HUMAN_unrelaxed_rank_001_alphafold2_multimer_v3_model_4_seed_000.pdb 71.56 9.81
b55_Q5T447-HECD3_HUMAN_unrelaxed_rank_002_alphafold2_multimer_v3_model_2_seed_000.pdb 71.27 27.62
b55_Q5T5U3-RHG21_HUMAN_unrelaxed_rank_001_alphafold2_multimer_v3_model_5_seed_000.pdb 0.0 8.52
b55_Q5T953-IER5L_HUMAN_unrelaxed_rank_001_alphafold2_multimer_v3_model_4_seed_000.pdb 90.5 2.03
b55_Q5T9S5-CCD18_HUMAN_unrelaxed_rank_001_alphafold2_multimer_v3_model_4_seed_000.pdb 0 30
b55_Q5VT52-RPRD2_HUMAN_unrelaxed_rank_001_alphafold2_multimer_v3_model_4_seed_000.pdb 0.0 8.1
b55_Q5VY09-IER5_HUMAN_unrelaxed_rank_002_alphafold2_multimer_v3_model_2_seed_000.pdb 90.91 2.43
b55_Q63ZY3-KANK2_HUMAN_unrelaxed_rank_003_alphafold2_multimer_v3_model_4_seed_000.pdb 56.69 13.2
b55_Q66LE6-2ABD_HUMAN_unrelaxed_rank_001_alphafold2_multimer_v3_model_1_seed_000.pdb 74.6 21.59
b55_Q6BDS2-BLT3A_HUMAN_unrelaxed_rank_005_alphafold2_multimer_v3_model_4_seed_000.pdb 80.91 26.42
b55_Q6NYC8-PPR18_HUMAN_unrelaxed_rank_001_alphafold2_multimer_v3_model_4_seed_000.pdb 0 30

Supplemental Table S1 continued. **AF2 scores for models with pLDDT and iPAE indicated after pdb file.**

b55_Q6NZY4-ZCHC8_HUMAN_unrelaxed_rank_001_alphafold2_multimer_v3_model_1_seed_000.pdb 0 30
b55_Q6P3X3-TTC27_HUMAN_unrelaxed_rank_003_alphafold2_multimer_v3_model_3_seed_000.pdb 74.7 28.36
b55_Q6P996-PDXD1_HUMAN_unrelaxed_rank_002_alphafold2_multimer_v3_model_2_seed_000.pdb 80.79 27.48
b55_Q6PID6-TTC33_HUMAN_unrelaxed_rank_001_alphafold2_multimer_v3_model_1_seed_000.pdb 73.23 25.44
b55_Q6UUV7-CRTC3_HUMAN_unrelaxed_rank_001_alphafold2_multimer_v3_model_5_seed_000.pdb 0 30
b55_Q70CQ4-UBP31_HUMAN_unrelaxed_rank_002_alphafold2_multimer_v3_model_4_seed_000.pdb 0.0 10.02
b55_Q70EL4-UBP43_HUMAN_unrelaxed_rank_001_alphafold2_multimer_v3_model_5_seed_000.pdb 63.92 9.12
b55_Q7RTV0-PHF5A_HUMAN_unrelaxed_rank_001_alphafold2_multimer_v3_model_2_seed_000.pdb 0.0 20.2
b55_Q7Z401-MYCPP_HUMAN_unrelaxed_rank_003_alphafold2_multimer_v3_model_1_seed_000.pdb 68.42 27.59
b55_Q7Z7K0-COXM1_HUMAN_unrelaxed_rank_002_alphafold2_multimer_v3_model_4_seed_000.pdb 59.97 22.11
b55_Q86SQ0-PHLB2_HUMAN_unrelaxed_rank_003_alphafold2_multimer_v3_model_4_seed_000.pdb 0.0 25.94
b55_Q86TC9-MYPN_HUMAN_unrelaxed_rank_003_alphafold2_multimer_v3_model_5_seed_000.pdb 0.0 21.66
b55_Q86U70-LDB1_HUMAN_unrelaxed_rank_001_alphafold2_multimer_v3_model_1_seed_000.pdb 0.0 24.52
b55_Q86UU1-PHLB1_HUMAN_unrelaxed_rank_002_alphafold2_multimer_v3_model_3_seed_000.pdb 73.11 13.88
b55_Q8IY67-RAVR1_HUMAN_unrelaxed_rank_004_alphafold2_multimer_v3_model_2_seed_000.pdb 0.0 23.84
b55_Q8N157-AHI1_HUMAN_unrelaxed_rank_001_alphafold2_multimer_v3_model_3_seed_000.pdb 80.41 13.94
b55_Q8N163-CCAR2_HUMAN_unrelaxed_rank_002_alphafold2_multimer_v3_model_2_seed_000.pdb 0 27.78
b55_Q8N5M4-TTC9C_HUMAN_unrelaxed_rank_001_alphafold2_multimer_v3_model_1_seed_000.pdb 75.21 17.14
b55_Q8N612-FHI1B_HUMAN_unrelaxed_rank_005_alphafold2_multimer_v3_model_2_seed_000.pdb 86.14 28.33
b55_Q8N8E3-CE112_HUMAN_unrelaxed_rank_003_alphafold2_multimer_v3_model_1_seed_000.pdb 67.59 26.52
b55_Q8NDG6-TDRD9_HUMAN_unrelaxed_rank_001_alphafold2_multimer_v3_model_4_seed_000.pdb 75.26 26.09
b55_Q8NHY2-COP1_HUMAN_unrelaxed_rank_001_alphafold2_multimer_v3_model_2_seed_000.pdb 83.5 10.85
b55_Q8TBC3-SHKB1_HUMAN_unrelaxed_rank_002_alphafold2_multimer_v3_model_3_seed_000.pdb 69.2 24.78
b55_Q8TCS8-PNPT1_HUMAN_unrelaxed_rank_002_alphafold2_multimer_v3_model_5_seed_000.pdb 0.0 27.75
b55_Q8TEP8-CE192_HUMAN_unrelaxed_alphafold2_multimer_v3_model_1_seed_000.pdb 68.18 27.75
b55_Q8TEU7-RPGF6_HUMAN_unrelaxed_rank_001_alphafold2_multimer_v3_model_1_seed_000.pdb 0.0 27.08
b55_Q8TF61-FBX41_HUMAN_unrelaxed_rank_001_alphafold2_multimer_v3_model_2_seed_000.pdb 68.55 8.16
b55_Q8WUF5-IASPP_HUMAN_unrelaxed_rank_001_alphafold2_multimer_v3_model_2_seed_000.pdb 60.77 9.71
b55_Q8WUI4-HDAC7_HUMAN_unrelaxed_rank_002_alphafold2_multimer_v3_model_1_seed_000.pdb 69.85 26.27
b55_Q8WUK0-PTPM1_HUMAN_unrelaxed_rank_001_alphafold2_multimer_v3_model_3_seed_000.pdb 82.57 4.2
b55_Q8WUM9-S20A1_HUMAN_unrelaxed_rank_001_alphafold2_multimer_v3_model_5_seed_000.pdb 79.37 3.87
b55_Q8WV74-NUDT8_HUMAN_unrelaxed_rank_001_alphafold2_multimer_v3_model_5_seed_000.pdb 64.39 12.93
b55_Q8WW01-SEN15_HUMAN_unrelaxed_rank_002_alphafold2_multimer_v3_model_1_seed_000.pdb 68.47 20.39
b55_Q8WXG6-MADD_HUMAN_unrelaxed_rank_001_alphafold2_multimer_v3_model_1_seed_000.pdb 0.0 28.11
b55_Q92526-TCPW_HUMAN_unrelaxed_rank_001_alphafold2_multimer_v3_model_4_seed_000.pdb 0 30
b55_Q92540-SMG7_HUMAN_unrelaxed_rank_002_alphafold2_multimer_v3_model_5_seed_000.pdb 64.35 15.07
b55_Q92771-DDX12_HUMAN_unrelaxed_rank_002_alphafold2_multimer_v3_model_4_seed_000.pdb 0.0 27.2
b55_Q96A73-P33MX_HUMAN_unrelaxed_rank_001_alphafold2_multimer_v3_model_3_seed_000.pdb 62.11 10.34
b55_Q96AT1-K1143_HUMAN_unrelaxed_rank_001_alphafold2_multimer_v3_model_4_seed_000.pdb 0 13.99
b55_Q96D53-COQ8B_HUMAN_unrelaxed_rank_003_alphafold2_multimer_v3_model_3_seed_000.pdb 0.0 23.81

Supplemental Table S1 continued. **AF2 scores for models with pLDDT and iPAE indicated after pdb file.**

b55_Q96D71-REPS1_HUMAN_unrelaxed_rank_002_alphafold2_multimer_v3_model_1_seed_000.pdb 0 13.81
b55_Q96DF8-ESS2_HUMAN_unrelaxed_rank_001_alphafold2_multimer_v3_model_1_seed_000.pdb 63.71 10.33
b55_Q96E09-PBIR1_HUMAN_unrelaxed_rank_001_alphafold2_multimer_v3_model_4_seed_000.pdb 82.4 2.99
b55_Q96FC9-DDX11_HUMAN_unrelaxed_rank_002_alphafold2_multimer_v3_model_4_seed_000.pdb 75.57 26.98
b55_Q96HB5-CC120_HUMAN_unrelaxed_rank_003_alphafold2_multimer_v3_model_3_seed_000.pdb 0 22.55
b55_Q96II8-LRCH3_HUMAN_unrelaxed_rank_001_alphafold2_multimer_v3_model_2_seed_000.pdb 72.13 9.43
b55_Q96IU2-ZBED3_HUMAN_unrelaxed_rank_003_alphafold2_multimer_v3_model_3_seed_000.pdb 0 25.5
b55_Q96N67-DOCK7_HUMAN_unrelaxed_rank_001_alphafold2_multimer_v3_model_1_seed_000.pdb 0 30
b55_Q96PE3-INP4A_HUMAN_unrelaxed_rank_001_alphafold2_multimer_v3_model_4_seed_000.pdb 82.04 25.95
b55_Q96QU8-XPO6_HUMAN_unrelaxed_rank_003_alphafold2_multimer_v3_model_2_seed_000.pdb 76.56 27.92
b55_Q96RT1-ERBIN_HUMAN_unrelaxed_rank_001_alphafold2_multimer_v3_model_4_seed_000.pdb 0 26.28
b55_Q99471-PFD5_HUMAN_unrelaxed_rank_001_alphafold2_multimer_v3_model_5_seed_000.pdb 0 30
b55_Q99504-EYA3_HUMAN_unrelaxed_rank_005_alphafold2_multimer_v3_model_4_seed_000.pdb 76.04 25.12
b55_Q99550-MPP9_HUMAN_unrelaxed_rank_002_alphafold2_multimer_v3_model_1_seed_000.pdb 59.99 11.63
b55_Q99832-TCPH_HUMAN_unrelaxed_rank_003_alphafold2_multimer_v3_model_3_seed_000.pdb 83.41 28.53
b55_Q99933-BAG1_HUMAN_unrelaxed_rank_002_alphafold2_multimer_v3_model_5_seed_000.pdb 65.63 20.67
b55_Q99958-FOXC2_HUMAN_unrelaxed_rank_003_alphafold2_multimer_v3_model_4_seed_000.pdb 75.17 8.7
b55_Q99959-PKP2_HUMAN_unrelaxed_rank_004_alphafold2_multimer_v3_model_3_seed_000.pdb 77.97 25.11
b55_Q9BQ95-ECSIT_HUMAN_unrelaxed_rank_003_alphafold2_multimer_v3_model_4_seed_000.pdb 73.54 25.25
b55_Q9BRK5-CAB45_HUMAN_unrelaxed_rank_002_alphafold2_multimer_v3_model_4_seed_000.pdb 71.53 25.22
b55_Q9BRQ8-FSP1_HUMAN_unrelaxed_rank_001_alphafold2_multimer_v3_model_4_seed_000.pdb 96.89 2.41
b55_Q9BSJ6-PIMRE_HUMAN_unrelaxed_rank_001_alphafold2_multimer_v3_model_3_seed_000.pdb 0 9.49
b55_Q9BSV6-SEN34_HUMAN_unrelaxed_rank_001_alphafold2_multimer_v3_model_2_seed_000.pdb 0 30
b55_Q9BTL4-IER2_HUMAN_unrelaxed_rank_002_alphafold2_multimer_v3_model_2_seed_000.pdb 89.46 2.4
b55_Q9BU02-THTPA_HUMAN_unrelaxed_rank_001_alphafold2_multimer_v3_model_3_seed_000.pdb 69.75 21.19
b55_Q9BU61-NDUF3_HUMAN_unrelaxed_rank_002_alphafold2_multimer_v3_model_1_seed_000.pdb 0 24.95
b55_Q9BUP3-HTAI2_HUMAN_unrelaxed_rank_003_alphafold2_multimer_v3_model_5_seed_000.pdb 62.21 18.39
b55_Q9BW92-SYTM_HUMAN_unrelaxed_rank_001_alphafold2_multimer_v3_model_5_seed_000.pdb 77.53 23.48
b55_Q9BWT7-CAR10_HUMAN_unrelaxed_rank_001_alphafold2_multimer_v3_model_4_seed_000.pdb 59.56 19.12
b55_Q9BXF6-RFIP5_HUMAN_unrelaxed_rank_003_alphafold2_multimer_v3_model_2_seed_000.pdb 55.96 17.81
b55_Q9BXL8-CDCA4_HUMAN_unrelaxed_rank_002_alphafold2_multimer_v3_model_4_seed_000.pdb 94.28 1.73
b55_Q9BYV8-CEP41_HUMAN_unrelaxed_rank_003_alphafold2_multimer_v3_model_2_seed_000.pdb 0.0 26.64
b55_Q9BZL6-KPCD2_HUMAN_unrelaxed_rank_002_alphafold2_multimer_v3_model_3_seed_000.pdb 64.39 25.06
b55_Q9C0B7-TNG6_HUMAN_unrelaxed_rank_001_alphafold2_multimer_v3_model_2_seed_000.pdb 0 30
b55_Q9H0K1-SIK2_HUMAN_unrelaxed_rank_001_alphafold2_multimer_v3_model_5_seed_000.pdb 82.48 3.58
b55_Q9H0R5-GBP3_HUMAN_unrelaxed_rank_002_alphafold2_multimer_v3_model_1_seed_000.pdb 0.0 26.39
b55_Q9H1H9-KI13A_HUMAN_unrelaxed_rank_005_alphafold2_multimer_v3_model_4_seed_000.pdb 66.54 26.64
b55_Q9H2J4-PDCL3_HUMAN_unrelaxed_rank_004_alphafold2_multimer_v3_model_3_seed_000.pdb 0.0 23.97
b55_Q9H4G0-E41L1_HUMAN_unrelaxed_rank_001_alphafold2_multimer_v3_model_5_seed_000.pdb 84.2 5.02
b55_Q9H4M9-EHD1_HUMAN_unrelaxed_rank_002_alphafold2_multimer_v3_model_1_seed_000.pdb 69.63 19.06

Supplemental Table S1 continued. **AF2 scores for models with pLDDT and iPAE indicated after pdb file.**

b55_Q9H5N1-RABE2_HUMAN_unrelaxed_rank_001_alphafold2_multimer_v3_model_3_seed_000.pdb 61.69 17.72
b55_Q9H5Z1-DHX35_HUMAN_unrelaxed_rank_001_alphafold2_multimer_v3_model_5_seed_000.pdb 76.47 12.42
b55_Q9H845-ACAD9_HUMAN_unrelaxed_rank_001_alphafold2_multimer_v3_model_5_seed_000.pdb 0.0 18.16
b55_Q9H910-JUPI2_HUMAN_unrelaxed_rank_001_alphafold2_multimer_v3_model_3_seed_000.pdb 0 11.65
b55_Q9HCN8-SDF2L_HUMAN_unrelaxed_rank_001_alphafold2_multimer_v3_model_3_seed_000.pdb 83.72 24.62
b55_Q9NNX1-TUFT1_HUMAN_unrelaxed_rank_001_alphafold2_multimer_v3_model_3_seed_000.pdb 77.25 9.48
b55_Q9NQ35-NRIP3_HUMAN_unrelaxed_rank_001_alphafold2_multimer_v3_model_1_seed_000.pdb 73.09 4.59
b55_Q9NQI0-DDX4_HUMAN_unrelaxed_rank_001_alphafold2_multimer_v3_model_4_seed_000.pdb 82.5 23.61
b55_Q9NQP4-PFD4_HUMAN_unrelaxed_rank_001_alphafold2_multimer_v3_model_3_seed_000.pdb 0 26.58
b55_Q9NQT8-KI13B_HUMAN_unrelaxed_rank_001_alphafold2_multimer_v3_model_4_seed_000.pdb 65.89 9.77
b55_Q9NR46-SHLB2_HUMAN_unrelaxed_rank_001_alphafold2_multimer_v3_model_5_seed_000.pdb 65.73 8.98
b55_Q9NRA8-4ET_HUMAN_unrelaxed_rank_001_alphafold2_multimer_v3_model_4_seed_000.pdb 69.39 7.13
b55_Q9NRL3-STRN4_HUMAN_unrelaxed_rank_001_alphafold2_multimer_v3_model_5_seed_000.pdb 0.0 25.8
b55_Q9NRV9-HEBP1_HUMAN_unrelaxed_rank_001_alphafold2_multimer_v3_model_3_seed_000.pdb 0.0 17.03
b55_Q9NSY1-BMP2K_HUMAN_unrelaxed_rank_001_alphafold2_multimer_v3_model_1_seed_000.pdb 64.61 13.47
b55_Q9NTX5-ECHD1_HUMAN_unrelaxed_rank_005_alphafold2_multimer_v3_model_2_seed_000.pdb 76.48 25.95
b55_Q9NUJ3-T11L1_HUMAN_unrelaxed_rank_002_alphafold2_multimer_v3_model_4_seed_000.pdb 61.26 26.64
b55_Q9NUP9-LIN7C_HUMAN_unrelaxed_rank_001_alphafold2_multimer_v3_model_1_seed_000.pdb 0.0 23.45
b55_Q9NZ71-RTEL1_HUMAN_unrelaxed_rank_001_alphafold2_multimer_v3_model_1_seed_000.pdb 69.82 12.29
b55_Q9NZW5-PALS2_HUMAN_unrelaxed_rank_003_alphafold2_multimer_v3_model_4_seed_000.pdb 0.0 24.89
b55_Q9P0K8-FOXJ2_HUMAN_unrelaxed_rank_001_alphafold2_multimer_v3_model_5_seed_000.pdb 77.34 9.42
b55_Q9P202-WHRN_HUMAN_unrelaxed_rank_001_alphafold2_multimer_v3_model_3_seed_000.pdb 64.86 9.99
b55_Q9P227-RHG23_HUMAN_unrelaxed_rank_002_alphafold2_multimer_v3_model_4_seed_000.pdb 58.69 13.43
b55_Q9P270-SLAI2_HUMAN_unrelaxed_rank_001_alphafold2_multimer_v3_model_4_seed_000.pdb 62.22 14.93
b55_Q9P2I0-CPSF2_HUMAN_unrelaxed_rank_004_alphafold2_multimer_v3_model_1_seed_000.pdb 69.28 27.06
b55_Q9UBI6-GBG12_HUMAN_unrelaxed_rank_001_alphafold2_multimer_v3_model_5_seed_000.pdb 0 30
b55_Q9UDT6-CLIP2_HUMAN_unrelaxed_rank_001_alphafold2_multimer_v3_model_4_seed_000.pdb 80.87 4.48
b55_Q9UDY4-DNJB4_HUMAN_unrelaxed_rank_001_alphafold2_multimer_v3_model_3_seed_000.pdb 73.09 27.41
b55_Q9UGU0-TCF20_HUMAN_unrelaxed_rank_001_alphafold2_multimer_v3_model_4_seed_000.pdb 0.0 9.26
b55_Q9UHB6-LIMA1_HUMAN_unrelaxed_rank_001_alphafold2_multimer_v3_model_4_seed_000.pdb 60.0 11.67
b55_Q9UJY1-HSPB8_HUMAN_unrelaxed_rank_003_alphafold2_multimer_v3_model_2_seed_000.pdb 0 25.83
b55_Q9UKY7-CDV3_HUMAN_unrelaxed_rank_002_alphafold2_multimer_v3_model_2_seed_000.pdb 0 11.74
b55_Q9UL15-BAG5_HUMAN_unrelaxed_rank_001_alphafold2_multimer_v3_model_4_seed_000.pdb 69.34 25.44
b55_Q9UNE7-CHIP_HUMAN_unrelaxed_rank_003_alphafold2_multimer_v3_model_1_seed_000.pdb 66.98 25.16
b55_Q9UPQ0-LIMC1_HUMAN_unrelaxed_rank_001_alphafold2_multimer_v3_model_5_seed_000.pdb 73.4 5.49
b55_Q9UPQ4-TRI35_HUMAN_unrelaxed_rank_001_alphafold2_multimer_v3_model_2_seed_000.pdb 72.63 27.3
b55_Q9UQL6-HDAC5_HUMAN_unrelaxed_rank_002_alphafold2_multimer_v3_model_2_seed_000.pdb 0.0 12.86
b55_Q9Y2H2-SAC2_HUMAN_unrelaxed_rank_001_alphafold2_multimer_v3_model_1_seed_000.pdb 0 14.95
b55_Q9Y2H5-PKHA6_HUMAN_unrelaxed_rank_002_alphafold2_multimer_v3_model_4_seed_000.pdb 68.3 8.59
b55_Q9Y2J4-AMOL2_HUMAN_unrelaxed_rank_001_alphafold2_multimer_v3_model_2_seed_000.pdb 64.32 11.27

Supplemental Table S1 continued. **AF2 scores for models with pLDDT and iPAE indicated after pdb file.**

b55_Q9Y2K2-SIK3_HUMAN_unrelaxed_rank_001_alphafold2_multimer_v3_model_1_seed_000.pdb 63.81 10.37
b55_Q9Y2Q3-GSTK1_HUMAN_unrelaxed_rank_001_alphafold2_multimer_v3_model_5_seed_000.pdb 69.74 22.84
b55_Q9Y2Z0-SGT1_HUMAN_unrelaxed_rank_003_alphafold2_multimer_v3_model_2_seed_000.pdb 0.0 25.17
b55_Q9Y371-SHLB1_HUMAN_unrelaxed_rank_001_alphafold2_multimer_v3_model_3_seed_000.pdb 64.44 19.0
b55_Q9Y375-CIA30_HUMAN_unrelaxed_rank_002_alphafold2_multimer_v3_model_2_seed_000.pdb 62.51 16.86
b55_Q9Y3X0-CCDC9_HUMAN_unrelaxed_rank_003_alphafold2_multimer_v3_model_5_seed_000.pdb 0 21.68
b55_Q9Y446-PKP3_HUMAN_unrelaxed_rank_001_alphafold2_multimer_v3_model_1_seed_000.pdb 0.0 11.04
b55_Q9Y4R8-TELO2_HUMAN_unrelaxed_rank_001_alphafold2_multimer_v3_model_4_seed_000.pdb 0 30
b55_Q9Y570-PPME1_HUMAN_unrelaxed_rank_002_alphafold2_multimer_v3_model_5_seed_000.pdb 75.52 5.41
b55_Q9Y580-RBM7_HUMAN_unrelaxed_rank_002_alphafold2_multimer_v3_model_1_seed_000.pdb 79.41 5.69
b55_Q9Y597-KCTD3_HUMAN_unrelaxed_rank_001_alphafold2_multimer_v3_model_2_seed_000.pdb 65.54 18.64
b55_Q9Y618-NCOR2_HUMAN_unrelaxed_alphafold2_multimer_v3_model_1_seed_000.pdb 66.88 6.69
b55_Q9Y6Q9-NCOA3_HUMAN_unrelaxed_rank_001_alphafold2_multimer_v3_model_4_seed_000.pdb 64.93 6.14

Supplemental Table S1 continued. AF2 scores for models with pLDDT and iPAE indicated after pdb file.

Table S2: Metrics of designed binders
Table S3: Mass spectrometry data
Table S4: Computational alanine scanning of B55 binders
Supplemental dataset 1: Alphafold models pdb files.
